# Effects of Soil Microbes on Functional Traits of Loblolly Pine (*Pinus taeda*) Seedling Families From Contrasting Climates

**DOI:** 10.3389/fpls.2019.01643

**Published:** 2020-01-09

**Authors:** Danielle E. M. Ulrich, Sanna Sevanto, Samantha Peterson, Max Ryan, John Dunbar

**Affiliations:** ^1^ Department of Ecology, Montana State University, Bozeman, MT, United States; ^2^ Earth and Environmental Sciences (EES-14), Los Alamos National Laboratory, Los Alamos, NM, United States; ^3^ Earth and Environmental Sciences Department, New Mexico Institute of Mining and Technology, Socorro, NM, United States; ^4^ Bioscience (B-11), Los Alamos National Laboratory, Los Alamos, NM, United States

**Keywords:** soil microbes, loblolly pine, seedling physiology, genetic variation, drought, turgor loss point, growth, root exudates

## Abstract

Examining factors that influence seedling establishment is essential for predicting the impacts of climate change on tree species’ distributions. Seedlings originating from contrasting climates differentially express functional traits related to water and nutrient uptake and drought resistance that reflect their climate of origin and influence their responses to drought. Soil microbes may improve seedling establishment because they can enhance water and nutrient uptake and drought resistance. However, the relative influence of soil microbes on the expression of these functional traits between seedling families or populations from contrasting climates is unknown. To determine if soil microbes may differentially alter functional traits to enhance water and nutrient uptake and drought resistance between dry and wet families, seeds of loblolly pine families from the driest and wettest ends of its geographic range (dry, wet) were planted in sterilized sand (controls) or in sterilized sand inoculated with a soil microbial community (inoculated). Functional traits related to seedling establishment (germination), water and nutrient uptake and C allocation (root:shoot biomass ratio, root exudate concentration, leaf C:N, leaf N isotope composition (δ^15^N)), and drought resistance (turgor loss point, leaf carbon isotope composition (δ^13^C)) were measured. Then, plants were exposed to a drought treatment and possible shifts in photosynthetic performance were monitored using chlorophyll fluorescence. Inoculated plants exhibited significantly greater germination than controls regardless of family. The inoculation treatment significantly increased root:shoot biomass ratio in the wet family but not in the dry family, suggesting soil microbes alter functional traits that improve water and nutrient uptake more so in a family originating from a wetter climate than in a family originating from a drier climate. Microbial effects on photosynthetic performance during drought also differed between families, as photosynthetic performance of the dry inoculated group declined fastest. Regardless of treatment, the dry family exhibited a greater root:shoot biomass ratio, root exudate concentration, and leaf δ^15^N than the wet family. This indicates that the dry family allocated more resources belowground than the wet and the two family may have used different sources of plant available N, which may be related to their contrasting climates of origin and influence their drought resistance. Examination of variation in impacts of soil microbes on seedling physiology improves efforts to enhance seedling establishment and beneficial plant-microbe interactions under climate change.

## Introduction

Increased intensity and frequency of heat waves, drought, and wildfire (IPCC, 2018) have led to widespread forest mortality in recent decades (Allen et al., 2010; Hartmann et al., 2018). To sustain forests for ecosystem services and atmospheric CO_2_ sequestration, it is essential to understand vegetation responses to changing climate that determine forest species’ geographic distributions under future climate regimes. Successful seedling establishment is a key determinant of future tree species’ distributions (Harper, 1977; Jackson et al., 2009; Zhu et al., 2012; Davis et al., 2016; Simeone et al., 2019). However, the seedling stage is the most vulnerable developmental stage of plants because seedlings are small and delicate with limited access to water and nutrients, which exacerbates their susceptibility to climate change stresses like drought (Johnson et al., 2011; García de la Serrana et al., 2015; Chen et al., 2016; Yao et al., 2018). Therefore, examining factors that influence seedling establishment and mortality (Kursar et al., 2009; Sapes et al., 2019) are crucial for predicting future species’ distributions.

Intraspecific (within-species) variation in the expression of functional traits related to water and nutrient uptake influences seedling establishment and physiological responses to drought (Sultan, 2000; Howe et al., 2003; Kawecki and Ebert, 2004; Isaac-Renton et al., 2018; Ramírez‐Valiente et al., 2018; Roches et al., 2018; Chauvin et al., 2019; Roskilly et al., 2019). Intraspecific adaptations to their climate of origin collectively enable a species to survive in diverse climates and span a large geographic range. As regions shift to more arid conditions under climate change, there is great research interest in identifying populations and families that will thrive under more arid conditions to facilitate adaptation and reforestation efforts (O’Neill et al., 2008; Grady et al., 2015; Moran et al., 2017). Provenance, greenhouse, and common garden studies have been used to examine how the differential expression of functional traits in seedling populations and families from contrasting climates influences physiological responses to drought. Populations and families from drier climates often exhibit functional traits that enable them to enhance water and nutrient uptake and resist drought more effectively than populations and families from wetter climates (Fernández et al., 1999; Cregg and Zhang, 2001; Nguyen-Queyrens and Bouchet-Lannat, 2003; Gratani, 2014; Kerr et al., 2015; Carvalho et al., 2017; Marias et al., 2017; Moran et al., 2017). Drought resistance is defined as the capacity of plants to avoid or tolerate drought, which is achieved through diverse physiological mechanisms (Levitt, 1980; Khanna-Chopra and Singh, 2015; Polle et al., 2019). Compared to seedling populations and families from wetter climates, seedling populations and families from drier climates can exhibit increased resource allocation to root growth which enhances water and nutrient uptake. Seedling populations and families from drier climates also can exhibit greater leaf carbon isotope ratios which indicate greater intrinsic water use efficiency and greater stomatal constraints on gas exchange, and lower leaf turgor loss point which can indicate greater drought tolerance (Grossnickle et al., 1996; Cregg and Zhang, 2001; Nguyen-Queyrens and Bouchet-Lannat, 2003; López et al., 2009; Bartlett et al., 2014; Kerr et al., 2015; Carvalho et al., 2017; Marias et al., 2017).

Soil microbial communities of bacteria and fungi have been suggested as a solution to improve seedling establishment because they can alter functional traits related to water and nutrient uptake and drought resistance (Kim et al., 2012). However, we do not know if and when soil microbial impacts on seedling function are positive or negative. Soil microbial communities and host-specific microbial associates can manipulate plant hormone signaling to stimulate root growth and water uptake (Bent et al., 2001; Vonderwell et al., 2001; Verbon and Liberman, 2016), increase nutrient availability to enhance nutrient uptake (Yang et al., 2009), and alter soil moisture conditions to delay the onset of drought (Gehring et al., 2017; Kannenberg and Phillips, 2017) and promote germination (Ulrich et al., 2019). Therefore, beneficial plant-microbe interactions can improve seedling establishment and adaptation to new conditions (Compant et al., 2010). However, soil microbes can also negatively influence plant function through pathogenesis and disease (Rodriguez et al., 2008; Mendes et al., 2013; Jacoby et al., 2017; Schirawski and Perlin, 2018). Furthermore, sustaining microbial symbionts comes at a significant C cost to plant hosts *via* the release of root exudates (Bais et al., 2006). The positive and negative impacts of soil microbes on seedlings, plus the overwhelming diversity of microbes in the soil, complicates prediction of soil microbial influences on plant physiological responses to drought (Allison and Martiny, 2008; Mendes et al., 2013; Finkel et al., 2017; Sánchez-Cañizares et al., 2017). Therefore, investigating how soil microbes influence seedling physiological response to drought in diverse systems is greatly needed (Dimkpa et al., 2009; de Zelicourt et al., 2013; Baldrian, 2017).

Given the ability of seedling populations and families from contrasting climates to differentially respond to drought, populations and families from contrasting climates may also differentially interact with soil microbes, which may influence plant water and nutrient acquisition. For example, drought tolerant and drought intolerant *Pinus edulis* associated with distinct ectomycorrhizal fungal communities in the field (Gehring et al., 2017). This suggests that microbial communities can vary by plant population, family, and genotype (Schweitzer et al., 2008; İnceoğlu et al., 2010; Berendsen et al., 2012; Whitham et al., 2012; Lamit et al., 2016) due to differential gene expression in functional traits such as the quantity and quality of root exudates used to shape the soil and root microbiome (Micallef et al., 2009; Mendes et al., 2013; Patel et al., 2015; Bakker et al., 2018). Variation in root exudate quantity and quality can attract beneficial microbes and repel harmful microbes to increase water and nutrient uptake (e.g., root growth) and drought resistance (Mendes et al., 2013; Coskun et al., 2017; Jacoby et al., 2017). One of the nutrients important to plant drought resistance is nitrogen (N) as numerous proteins underlie plant functional responses to stress. RUBISCO is the main protein that determines photosynthetic capacity, and thus contributes to the ability to maintain gas exchange during stress (Field and Mooney, 1986; Evans, 1989). Drought stress can inhibit RUBISCO activity *via* reductions in ribulose-1,5-bisphosphate (RuBP) (Gimenez et al., 1992) and reductions in the large subunit of RUBISCO (Majumdar et al., 1991). Different soil microbes are responsible for converting organic N into forms that are accessible to plants: either ammonium (NH_4_^+^) or nitrate (NO_3_^-^) (Hayatsu et al., 2008; Ohyama, 2010; Jacoby et al., 2017). Therefore, within-species populations and families can use root exudates to recruit and repel different soil microbes (Haney et al., 2015; Wille et al., 2019) that cause the plant populations and families to use different forms of N (i.e., NH_4_^+^, NO_3_^-^). The form of N used by the plant is reflected in leaf N isotope ratios (δ^15^N) (Kahmen et al., 2008; Temperton et al., 2012). Therefore, within-species variation in functional traits like leaf δ^15^N, %N, and root exudates suggests that within-species populations and families may differentially interact with soil microbes, altering nutrient acquisition among populations and families.

Intraspecific variation in plant-microbe interactions suggest that soil microbes may differentially affect the performance of plant populations and families under drought by differentially altering functional traits related to water and nutrient uptake and drought resistance. Indeed, the direction and magnitude of the effects of soil microbes on plant performance can vary among within-species groups (O’Brien et al., 2018). For example, during drought, *Ostrya virginiana* and *Betula nigra* seedling populations grew more biomass when grown with soil microbes originating from drier sites than when grown with soil microbes from wetter sites (Allsup and Lankau, 2019). This suggests dry-adapted soil microbes may drive greater improvements in plant productivity in populations and families from wetter climates than drier climates. However, this hypothesis has only been tested on plant biomass and needs to be tested on functional traits more directly related to water and nutrient uptake and drought resistance in diverse systems. Extending this research beyond just plant biomass and focusing on functional traits that elucidate the impact of soil microbes on plant physiology improves efforts to engineer beneficial plant-microbe interactions under climate change. Identifying specific plant populations and families that may gain the greatest physiological benefits from soil microbes facilitates seedling adaptation and reforestation efforts.

Loblolly pine (*Pinus taeda* L.) is the most widely planted and the most economically valuable species in the southern USA (Schultz, 1997). Its geographic distribution spans a wide range of moisture conditions from its driest edge in eastern Texas to its wettest edge on the mid-Atlantic coast, USA (97.5W to 75.0W). Studies have shown that loblolly pine populations and families can vary in functional traits related to drought resistance including growth (Sierra-Lucero et al., 2002) and physiology, where populations and families from drier locations were often, but not always, more drought resistant (Buijtenen et al., 1976; Wells, 1983; Lambeth et al., 1984; but see (Bongarten and Teskey, 1986; Meier et al., 1992; Yang et al., 2002). Mycorrhizal fungi can improve water and nutrient uptake in loblolly pine (Ford et al., 1985; Constable et al., 2001) and plant growth promoting bacteria can have both positive and negative effects on loblolly pine seedling growth (Enebak et al., 1998).

Here, our objective was to determine if and how soil microbes differentially influence functional traits and photosynthetic performance under drought in loblolly pine families from contrasting climates. We grew seeds of loblolly pine families from the driest and wettest ends of its geographic range (dry, wet) and inoculated seeds of both families with a dry-adapted soil microbial community (inoculated). We measured functional traits related to seedling establishment (germination), water and nutrient uptake, and carbon allocation (root:shoot biomass ratio, root exudate concentration, leaf C:N, leaf N isotope composition), and drought resistance (turgor loss point, leaf C isotope composition) before drought. We imposed a drought by completely withholding water and monitored photosynthetic performance using chlorophyll fluorescence. We hypothesized that soil microbes would differentially alter functional traits between the dry and wet families where soil microbes would alter functional traits to enhance water and nutrient uptake and drought resistance to a greater extent in a family originating from a wetter climate than in a family originating from a drier climate.

## Materials and Methods

### Plant Material and Experimental Set-Up

To test our hypothesis, we used a controlled greenhouse experiment where loblolly pine seedlings from dry and wet climates of origin were grown from seed in sterilized sand and exposed to an inoculation treatment with a soil microbial community from an arid region. Single family, open-pollinated, geographically distinct (Schultz, 1997) loblolly pine seed originating from Bastrop county in Texas (“dry”) and Orangeburg county in South Carolina (“wet”) were provided by the Western Gulf Forest Tree Improvement Program and International Forest Genetics & Seed Company, respectively. These families originated from climates that represent loblolly pine’s wettest and driest ends of its geographic range (Schultz, 1997). Mean annual precipitation of the wet family’s climate of origin was 33% greater than that of the dry family (**Table 1**; PRISM, 2018).

**Table 1:** Location and climate information (1960–2017) for the dry and wet families and the drought-adapted soil microbial community used for inoculation treatment.

	Dry	Wet	Soil microbial community
**Region**	Interior Texas, USA (“lost pines”)	Coastal South Carolina, USA	Interior New Mexico, USA
**Latitude (°N)**	30.1036	33.4390	35.5194
**Longitude (°W)**	−97.3120	−80.8003	−106.2277
**Elevation (m)**	131	54	1750
**MAT (°C)**	20.0	17.8	12.1
**T_min_**	13.5	11.3	3.8
**T_max_**	26.4	17.8	20.5
**MAP (mm)**	917	1215	306
**MVPD (hPa)**	10.9	9.2	12.2

Mean annual temperature (MAT), minimum temperature (T_min_), maximum temperature (T_max_), mean annual precipitation (MAP), mean vapor pressure deficit (MVPD).

Inoculated plants were inoculated with a microbial community from a soil sample collected 0–5 cm deep in north central New Mexico, USA (35.5194, −106.2277), a site that receives 67% less mean annual precipitation than the dry family and 75% less mean annual precipitation than the wet family (**Table 1**). Because we aimed to focus on how seedling families from contrasting climates may differentially interact with any kind of soil microbial community, we selected a soil microbial community outside the contemporary range of loblolly pine. By choosing a soil microbial community to which neither family has been exposed enabled us to isolate contrasting seedling family responses to a novel microbial community, not confounded by previous exposure to such a microbial community. The microbial community was sequenced as in (Ulrich et al., 2019) to determine its bacterial and fungal composition. Briefly, DNA extractions were performed using the DNeasy PowerSoil DNA extraction kit (Qiagen, Hilden, Germany). DNA samples were quantified with an Invitrogen Quant-iTTM ds DNA Assay Kit on a BioTek Synergy HI Hybrid Reader. PCR templates were prepared by diluting an aliquot of each DNA stock in sterile water to 1 ng/μl. The bacterial (and archaeal) 16 S rRNA gene (V3-V4 region) was amplified using primers 515f-806r (Mueller et al., 2016). The fungal 28 S rRNA gene (D2 hypervariable region) was amplified using the LR22R and the reverse LR3. Amplicons were cleaned using a Mobio UltraClean PCR clean-up kit, quantified using the same procedure as for the extracted DNA, and then pooled at a concentration of 10 ng each. A bioanalyzer was used to assess DNA quality, concentration was verified using qPCR, and paired-end 250 bp reads were obtained using an Illumina MiSeq sequencer at the Los Alamos National Laboratory, NM, USA.

To create the soil inoculum for the microbial inoculation treatment, microbes from the soil were extracted by suspension in sterile DI water to create a 1:20 dilution; this isolates the microbial community and reduces potential biogeochemical effects of the original soil. Before planting, stratified seeds (moistened then stored at 35°C for 45 days) were surface sterilized for 10 min in 10% bleach and rinsed for 15 min in sterile DI water twice. Seeds were then soaked in the soil inoculum (inoculated) or sterile DI water (controls) for 10 min. Seeds were each planted in 2.65 L pots (10.2 cm × 10.2 cm × 34.3 cm) of sterilized sand (1 seed per pot) on 23 August 2017 (day 0). Five ml of soil inoculum (inoculated) or sterile DI water (controls) was applied to each pot once during initial planting and also a second time 13 days (5 September 2017) after planting to ensure effects of soil microbial communities (e.g., Corkidi et al., 2002). Both the soil inoculum and water-only treatments each were applied to 15 pots that formed the inoculated and control groups, respectively (N = 15 in each group and family). Clear plastic was placed over all pots to maintain high humidity to promote germination and then was removed after 13 days when most of the plants had germinated. A fertilizer treatment of 5 ml of ammonium nitrate (1 mg/ml) was applied evenly to each pot 19 days after planting (11 September 2017). All plants were well-watered every 2–3 days to field capacity using reverse osmosis (RO) water filtered with a 0.2-µm filter, until drought treatment during which water was completely withheld. Drought was imposed by completely withholding water beginning on day 395 after planting (22 September 2018).

Plants were grown under a 14-h photoperiod in a temperature-controlled greenhouse at the New Mexico Consortium in Los Alamos, New Mexico, USA. Average daytime temperature in the greenhouse was 22.6°C, average nighttime temperature 20.6°C, average daytime relative humidity 47.5%, and average daily maximum photosynthetic photon flux density (PPFD) was 382.4 umol m^-2^ s^-1^.

### Measurements

Germination was determined by counting the total number of individuals that germinated per group (inoculated, control) in each family (dry, wet) 14 days after planting (6 September 2017). Shoot height of all 15 individuals per group in each family was measured 287 days after planting (6 June 2018).

To examine family and treatment effects on gas exchange, photosynthesis (*A*) and stomatal conductance (*g*_s_) were measured on four randomly selected individuals per group in each family using a portable photosynthesis system with an infrared gas analyzer (LI-6400 XT, Licor, Lincoln, NE, USA) on day 307 (26 June 2018) between 08h00 and 11h00. In the cuvette, flow rate was set to 500 µmol s^-1^, reference [CO_2_] 400 µmol mol^-1^, quantum flux 2,000 µmol m^-2^ s^-1^ to avoid any light limitation of *A*, and leaf temperature 20°C. Needles in the cuvette (eventually collected for biomass measurements; see below) were scanned using ImageJ image processing software (Schindelin et al., 2012; Schneider et al., 2012) to determine leaf area and normalize gas exchange values by leaf area.

After gas exchange was measured, root exudate concentration was measured in the same four individuals per group in each family used for gas exchange measurements. Total organic carbon (TOC) released by roots (hereafter referred to as root exudates) was collected using methods adapted from (Phillips et al., 2008; Karst et al., 2017; Preece et al., 2018). Loblolly seedlings were carefully dug up to keep roots intact, and roots were dipped in an antimicrobial solution (10,000 units penicillin, 10 mg streptomycin, and 25 μg amphotericin B per ml) to halt microbial activity. Seedlings were then transplanted into pots of glass beads (500-µm diameter). Pots were wrapped in aluminum foil to exclude light and seedlings were allowed to acclimate for 3 days. For root exudate collection, seedlings were first flushed with 150 ml of sterile DI H_2_O using a vacuum pump. Another 150 ml of sterile DI H_2_O was added and seedlings were left to release exudates for 24 h. Root exudates were then collected in vials with a vacuum pump. TOC concentration of the collected root exudates was measured using a microplate reader with a UV-visible absorbance detector (Synergy H1 Hybrid Reader, BioTek, Winooski, VT, USA) at a wavelength of 254 nm. Root exudate concentration was determined by converting absorbance (x) to concentration (y) using the linear relationship: y = 6.3259x + 2.5901 (R^2^^=^ 0.6). This relationship was determined using 92 liquid TOC samples that had been measured with both UV-visible absorbance and a wet oxidation TOC analyzer (OI Analytical model 1010, Xylem Inc., Rye Brook, NJ, USA) as in (Jones et al., 2014). This method is advantageous because soil water TOC concentrations can be rapidly and cheaply estimated from spectral properties, yet may be limited because variability in concentration within a soil type was observed (Jones et al., 2014). Root exudate concentration values were normalized by dry root biomass. Roots and shoots from the same four plants per group in each family were then harvested, dried, and weighed for root:shoot biomass measurements. While roots were excavated for root exudate collection, we observed that seedlings were not root bound.

To evaluate intraspecific differences in the effect of inoculation treatment on nutrient uptake and drought resistance, dried leaves from the same four plants per group in each family were then analyzed for C and N content (%) and C and N isotope ratios (δ^13^C, δ. Approximately 0.8 mg of dried needle powder was packed in tin capsules for combustion for subsequent elemental analysis using a stable isotope ratio mass spectrometer (Finnigan MAT253, Thermo Electron Corporation, Waltham, MA, USA) coupled to an elemental analyzer (Costech Analytical Technologies, Inc., Valencia, CA, USA). The δ and^15^N were recorded as deviations per thousand (‰) and were calibrated using International Atomic Energy Agency (IAEA) standards C3, C6, C8, N1, and N2. The δ^13^C of leaf tissue (δ^13^C_leaf_) reflects the δ^13^C of CO_2_ in the atmosphere (δ^13^C_air_), the fractionation against the heavier carbon isotope (^13^C) due to physiological processes and the ratio of the concentration of CO_2_ inside the leaf (*c*_i_) to that in the ambient air (*c*_a_):

(1) $${\text{δ}}^{13}{\text{C}}_{leaf}={\text{δ}}^{13}{\text{C}}_{air}-a-\left(b-a\right)\frac{c_{i}}{c_{a}}$$

where *a* is the fractionation effect of diffusion of CO_2_ through stomata (4.4‰), and *b* is the fractionation effect (27‰) associated with discrimination against ^13^C by the enzyme RUBISCO during carbon fixation (Farquhar et al., 1982; Farquhar and Richards, 1984). δ^13^C_leaf_ is also an integrated measure of intrinsic water-use efficiency (iWUE) at the time the tissue was formed where greater δ^13^C_leaf_ (i.e., less negative) indicates greater iWUE (Farquhar et al., 1989). Like δ^13^C_leaf_, the δ^15^N of leaf tissue reflects sources of N taken up by the plant and the possible discrimination against ^15^N during the assimilation of each source (Shearer and Kohl, 1986). Discrimination for the reduction of nitrate to nitrate can occur *via* the nitrate reductase enzyme (Comstock, 2001; Cernusak et al., 2009) where *a* is the fractionation effect of diffusion of CO_2_ through stomata (4.4‰), and *b* is the fractionation effect (27‰) associated with discrimination against ^13^C by the enzyme RUBISCO during carbon fixation (Farquhar et al., 1982; Farquhar and Richards, 1984). δ^13^C_leaf_ is also an integrated measure of intrinsic water-use efficiency (iWUE) at the time the tissue was formed where greater δ^13^C_leaf_ (i.e., less negative) indicates greater iWUE (Farquhar et al., 1989). Like δ^13^C_leaf_, the δ^15^N of leaf tissue reflects sources of N taken up by the plant and the possible discrimination against ^15^N during the assimilation of each source (Shearer and Kohl, 1986). Discrimination for the reduction of nitrate to nitrate can occur *via* the nitrate reductase enzyme (Comstock, 2001; Cernusak et al., 2009).

Before drought treatment, we measured leaf drought tolerance using the water potential at turgor loss or turgor loss point (Ψ_TLP_; Bartlett et al., 2014) on four new individuals randomly selected per group (inoculated, control) in each family (dry, wet; 16 curves total) not used for the aforementioned measurements. Ψ_TLP_ was determined from pressure-volume (*P*-*V*) curves as in (Meinzer et al., 2014). P-V curves plot leaf water potential in response to changes in water volume as leaves dry and are used to determine bulk leaf parameters related to leaf cellular composition and structural properties such as Ψ_TLP_. Species with lower (more negative) Ψ_TLP_ are more tolerant of drought because they are able to maintain turgor and function under more negative soil water potentials (Bucci et al., 2004; Blackman et al., 2010). *P*-*V* curves were measured over five days (days 338-343, 27 July–1 August 2018). Seedlings were cut at predawn and were then placed in beakers of water to rehydrate for 2-3 hours. No rehydration-induced plateau was detected (Kubiske and Abrams, 1991). Shoots were allowed to dry out slowly on the laboratory bench. Measurements of shoot mass taken with a balance, and water potential taken with a pressure chamber (PMS Instruments, Albany, OR, USA) were recorded as shoots dried out. Data were plotted with relative water deficit on the x-axis and 1/Ψ on the y-axis. Data were checked during measurement to ensure at least 3–5 points were recorded along the linear portion of the curve. Ψ_TLP_ was estimated from the intersection of the linear portion of the curve with a negative exponential function fitted to the nonlinear portion as in (Meinzer et al., 2014).

To compare the effects of drought on inoculated and control groups in both families, drought was imposed by completely withholding water beginning on day 395 after planting (22 September 2018). To detect family and treatment differences in drought effects on seedling physiology related to photosynthetic performance, dark-adapted chlorophyll fluorescence was measured on the remaining seven individuals per group in each family the day before drought began and then weekly until photosynthetic performance declined to zero. Chlorophyll fluorescence measurements were made on mature, fully expanded needles at ambient temperature using a field portable pulse-modulated chlorophyll fluorometer (FMS2, Hansatech, Norfolk, UK) at predawn to ensure plants were dark-adapted. Chlorophyll fluorescence was measured as the ratio of variable to maximum fluorescence (F_V_/F_M_) in the convention of Maxwell and Johnson (2000). F_V_/F_M_ measures the maximum quantum efficiency of PSII photochemistry (Genty et al., 1989) and is calculated as:

$$\frac{F_{V}}{F_{M}}=\frac{F_{M}-F_{O}}{F_{M}}=1-\;\frac{F_{O}}{F_{M}},$$

where F_V_ is variable fluorescence, F_M_ is maximum fluorescence, and F_O_ is the minimum level of fluorescence. F_O_ was induced using a measuring light (red light-emitting diode, 650 nm, 0.15 μmol m^-2^ s-^1^ PAR) with a pulse-width of 3 μs and a pulse modulation frequency of 0.6 kHz. F_V_/F_M_ was then determined by applying a 0.8 s saturating pulse of white light (18,000 μmol photons m^-2^ s-^1^ PAR), which transiently closed all PSII reaction centers (preventing any photochemical processes from occurring), minimized heat dissipation (since leaves were dark-adapted), and induced maximum and variable fluorescence. F_V_/F_M_ is considered a sensitive indicator of photosynthetic performance (Maxwell and Johnson, 2000) and has been used to monitor photosynthetic function during severe drought stress (e.g. Ings et al., 2013; García de la Serrana et al., 2015; Chen et al., 2016; Yao et al., 2018). Because F_V_/F_M_ is most affected by severe drought, we focused on relative F_V_/F_M_ differences among groups under severe drought. Optimal F_V_/F_M_ values are ~0.8 (Björkman and Demmig, 1987); however, our plants before drought had F_V_/F_M_ values ~0.6 because plants were grown in pure, fast-draining sand with low nutrients. F_V_/F_M_ before drought did not significantly differ among families or treatment (P > 0.05). We used F_V_/F_M_ as a rapid, nondestructive method to compare relative differences in effects of severe drought among groups. We did not measure gas exchange because it requires destructively harvesting the leaf area (to correct gas exchange measurements for leaf area) and we did not want needle loss to influence the functions of the remaining needles (for example, compensatory gas exchange that can occur in response to defoliation).

### Statistical Analyses

A two-way ANOVA was used to evaluate the significance of the main effects of family and treatment on functional traits (i.e. response variables): germination, height, root:shoot biomass ratio, root exudates, turgor loss point, leaf %C, %N and C:N, leaf δ^13^C and δ^15^N, photosynthesis (*A*), and stomatal conductance (*g*_s_). Assumptions of equal variance and normality were checked using residual and quantile-quantile plots. Root:shoot biomass was log-transformed to meet the normality assumption. Tukey’s posthoc test was used to identify statistically significant differences in means. To compare differences in inoculation treatment effects between families, we determined the treatment effect size and the 95% confidence interval on the aforementioned functional traits for both the dry and wet families. Effect size was calculated as the mean difference in functional trait between the control and inoculated groups divided by the pooled standard deviation within each family. We corrected the effect size for bias using the standard correction that accounts for small sample averages according to (Hedges et al., 1985; Lakens, 2013; Hedges and Olkin, 2014). Effect size was considered to be significant if the 95% confidence interval did not overlap with an effect size of 0. Effect size was considered to be moderately significant if the 95% confidence interval overlapped an effect size of 0 by less than ± 0.1. A positive effect size indicates an increase in the functional trait.

A linear mixed effects model was used to determine differences in F_V_/F_M_ between inoculated and control groups through time during the drought treatment. Fixed effects were treatment (inoculated, control), family (dry, wet), and week and the random effect was individual. A linear mixed effects model, as opposed to the two-way ANOVA, enables us to account for repeated measurements through time by fitting models with different correlation structures. The model of best fit was selected based on Akaike information criterion (AIC) values. Assumptions of constant variance and normality were checked using residual and quantile-quantile plots. All interactive and main effects of factors on the response were tested using marginal F-tests (also known as type III tests). Post-hoc comparisons were made using a 95% confidence interval and P ≤ 0.05. All statistical analyses were conducted in R version 3.4.2 (R Core Team, C. T., 2018). The linear mixed effects model was conducted using the *nlme* and *gmodels* R packages (Warnes et al., 2018; Pinheiro et al., 2019).

## Results

**Figure 1:**
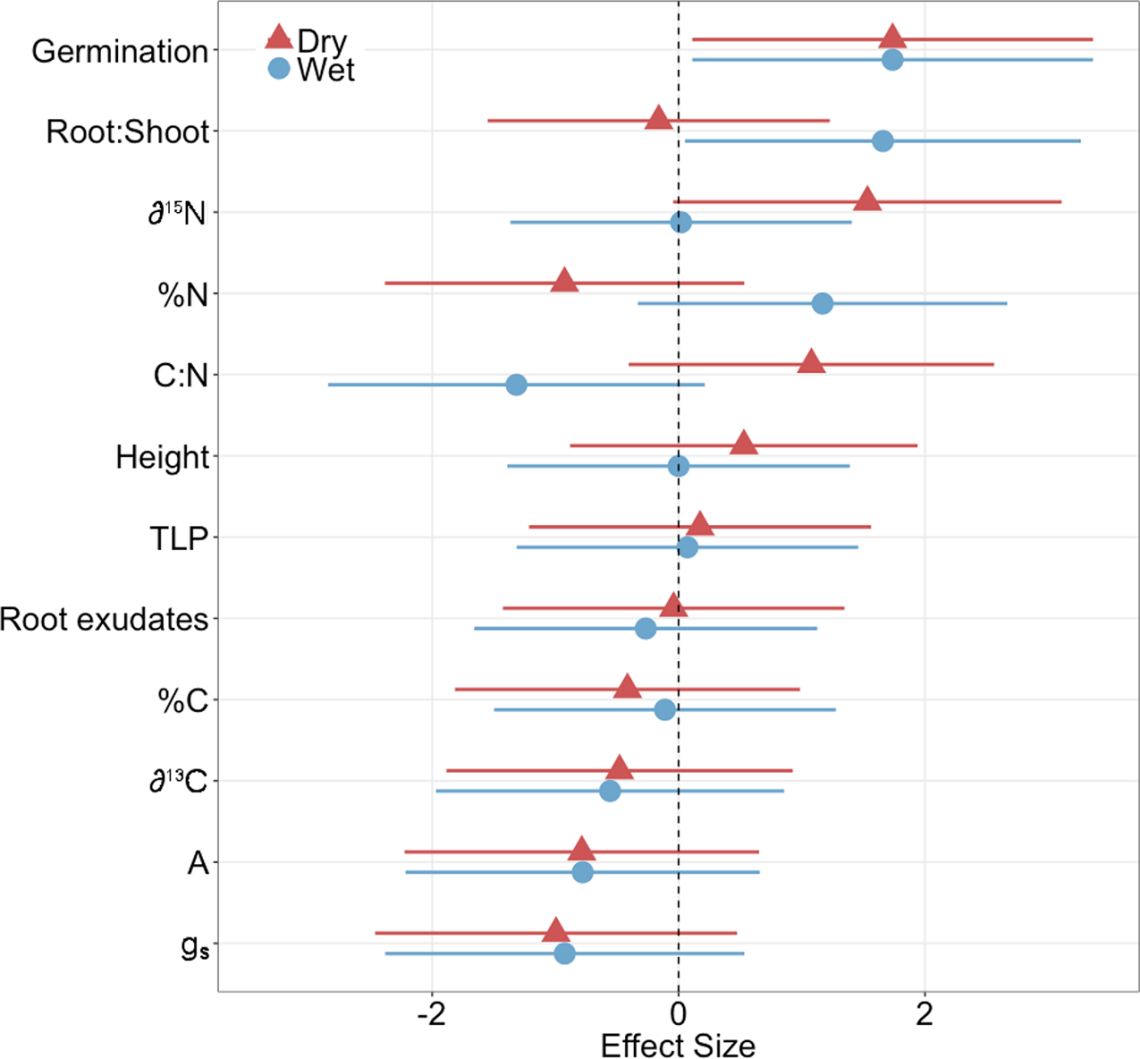
Effect size of inoculation treatment on physiological measurements in dry and wet families (germination, root:shoot biomass ratio, leaf N isotope ratio (δ^15^N), leaf N content (%N), leaf C:N ratio (C:N), height, turgor loss point (Ψ_TLP_), root exudate concentration, leaf C content (%C), leaf C isotope ratio (δ^13^C), photosynthesis (A), stomatal conductance (g_s_)). A positive effect size indicates an increase in the physiological measurement. Bars represent 95% confident intervals. Effect sizes were considered significant if the 95% confidence intervals did not overlap with an effect size of zero. N = 4. Seedlings were ~11 months old for all measurements except germination (14 days old) and height (~9 months old).

**Figure 2:**
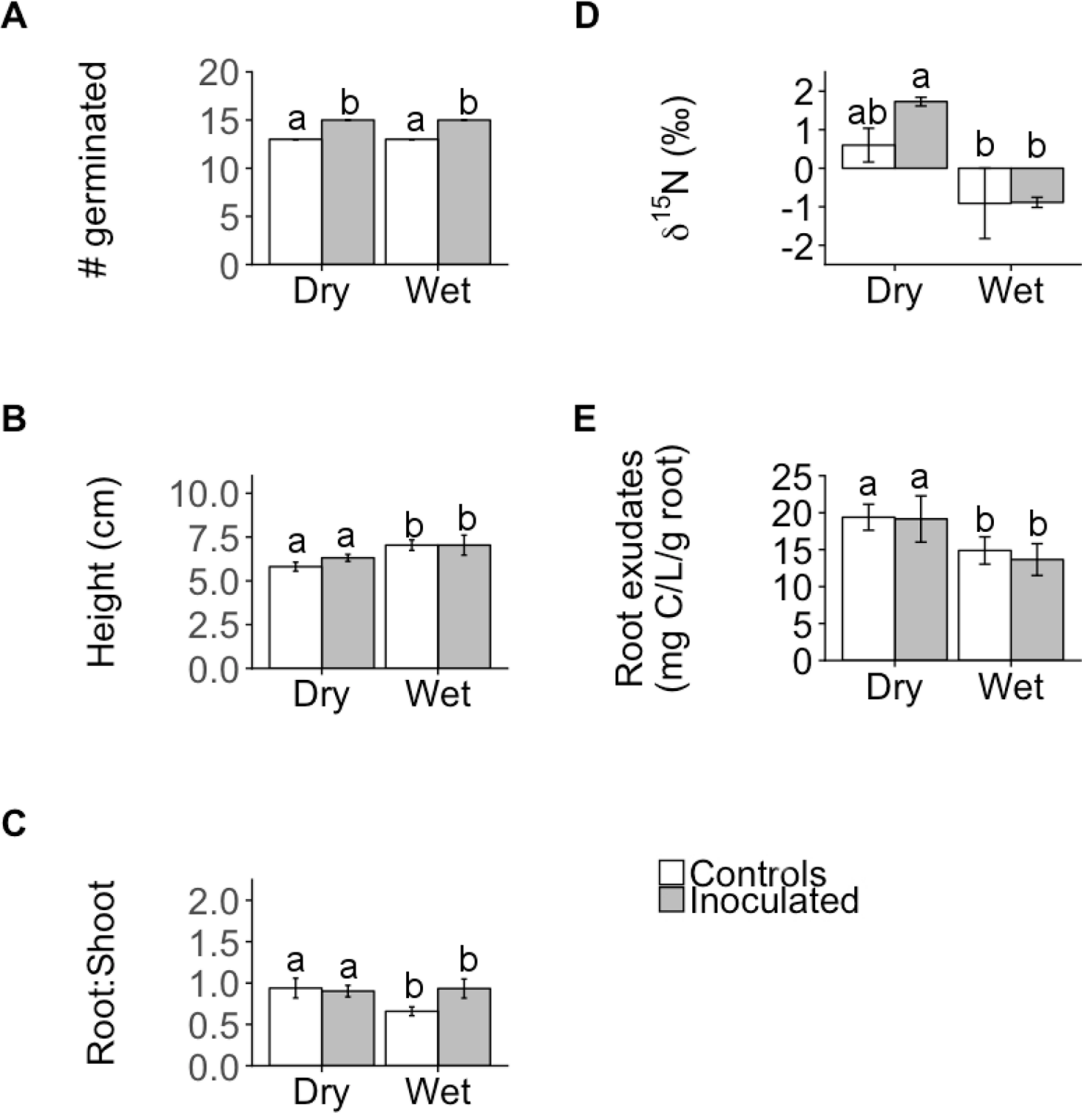
Mean number of individuals germinated **(A)**, shoot height **(B)**, root:shoot biomass ratio (root:shoot; **C**), leaf N isotope ratios (δ^15^N; **D**), and root exudate concentration **(E)** of control and inoculated groups in dry and wet families. Letters indicate statistically significant differences among the four groups at P ≤ 0.05. Error bars represent ± SE. N = 4. Seedlings were ~11 months old for all measurements except germination (14 days old) and height (~9 months old).

The microbial inoculation treatment affected functional traits of both families similarly for the majority of traits we measured (germination, height, Ψ_TLP_, root exudate concentration, %C, δ^13^ C, photosynthesis (*A*), and stomatal conductance (*g*_s_)) but the effect size was only significant for one trait: germination (**Figure 1**, **Supplementary Table 1**), where inoculated plants exhibited significantly greater germination than controls regardless of family (ANOVA; P < 0.001; **Figures 1** and **2A**, **Supplementary Table 2**). In contrast, the inoculation treatment affected the remaining traits (root:shoot biomass ratio, leaf δ^15^N, %N, C:N) of the dry and wet families in opposite directions but the effect size was only significant or moderately significant for two traits: root:shoot biomass ratio and leaf δ^15^N (**Figure 1**, **Supplementary Table 1**). The inoculation treatment significantly increased the root:shoot biomass ratio in the wet family but not the dry family, as indicated by only the wet family’s significant, positive effect size of 1.7. The inoculation treatment increased leaf δ^15^N in the dry family but not the wet family, as indicated by only the dry family’s moderately significant, positive effect size of 1.5 (i.e., 95% CI = −0.05 to 3.1, **Supplementary Table 1**). Despite these significant and moderately significant effect sizes on root:shoot biomass ratio and leaf δ^15^N, the ANOVA did not result in significant effects of treatment or the interaction on root:shoot biomass ratio and leaf δ^15^N in either family (ANOVA; P = 0.78, 0.12; P = 0.30, 0.23; **Figures 2C, D**, **Supplementary Table 2**), possibly due to sample size (N = 4).

Despite the variation in the direction and magnitude of treatment effect size on measured functional traits between plant families, the dry family exhibited functional traits indicative of a greater capacity to withstand drought than the wet family regardless of treatment. Regardless of treatment, the dry family exhibited a significantly greater root:shoot biomass ratio (P = 0.047; **Figure 2C**), was significantly shorter (P = 0.030, **Figure 2B**), exhibited significantly greater leaf δ^15^N (P < 0.001, **Figure 2D**), and released a greater root exudate concentration compared to the wet family (P = 0.031, **Figure 2E**).

Leaf δ^13^C, %C, %N, C:N, *A*, *g*_s_, and Ψ_TLP_ were not significantly influenced by treatment, family, or the interaction between treatment and family (P > 0.05; **Table 2**, **Supplementary Table 2**). Germination was not affected by family or the interaction between treatment and family (P = 0.27, 0.55, respectively; **Figure 2A**, **Supplementary Table 2**). Height was not significantly affected by treatment or the interaction (P = 0.35, 0.51, respectively; **Figure 2B**, **Supplementary Table 2**). Root exudate concentration was not affected by treatment or the interaction (P = 0.19, 0.080, respectively; **Figure 2E**, **Supplementary Table 2**). In addition to Ψ_TLP_, other parameters derived from pressure-volume curves were not significantly influenced by treatment, family, or the interaction between treatment and family (P > 0.05; **Supplementary Table 3**). Representative pressure-volume curves for each family and treatment are included in **Supplementary Figure 1**.

**Table 2:** Physiological measurements of control and inoculated groups in dry and wet families (leaf C content, N content, C:N ratio, C isotope ratio (δ^13^C), photosynthesis (*A*), stomatal conductance (*g*_s_), turgor loss point (Ψ_TLP_)).

	Dry		Wet	
	Controls	Inoculated	Controls	Inoculated
**C content** **(%)**	45.42 ± 0.28 a	45.10 ± 0.39 a	45.77 ± 0.46 a	45.67 ± 0.39 a
**N content** **(%)**	0.50 ± 0.062 a	0.41 ± 0.0090 a	0.40 ± 0.011 a	0.47 ± 0.036 a
**C:N**	93.52 ± 9.0 a	110.41 ± 3.41 a	114.88 ± 2.06 a	98.84 ± 7.3 a
**δ^13^C** **(‰)**	−29.9 ± 0.8 a	−30.5 ± 0.4 a	−29.8 ± 0.2 a	−30.5 ± 0.7 a
***A*** **(µmol m^-2^ s^-1^)**	1.67 ± 0.83 a	0.74 ± 0.078 a	1.37 ± 0.63 a	0.82 ± 0.069 a
***g*_s_** **(mol m^-2^ s^-1^)**	0.038 ± 0.008 a	0.018 ± 0.002 a	0.033 ± 0.008 a	0.030 ± 0.006 a
**Ψ_TLP_** **(MPa)**	−1.36 ± 0.08 a	−1.34 ± 0.07 a	−1.36 ± 0.11 a	−1.34 ± 0.12 a

Letters indicate statistically significant differences among groups at P ≤ 0.05. All values are expressed as means ± SE. N = 4. Seedlings were ~11 months old.

**Figure 3:**
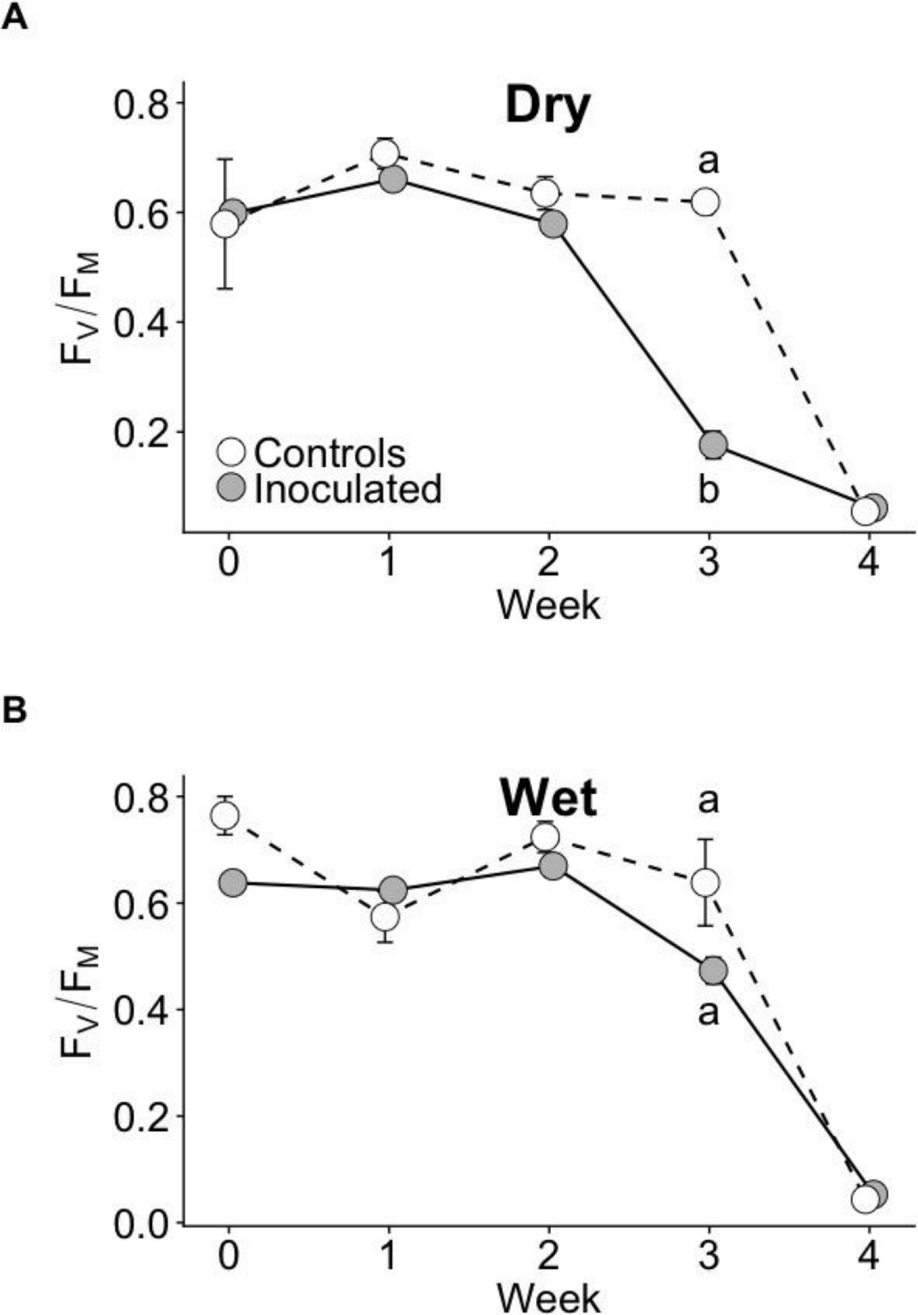
F_V_/F_M_ time courses of control and inoculated groups in dry **(A)** and wet **(B)** families measured weekly before and throughout drought. Drought was imposed after week 0 (when seedlings were ~13 months old). Error bars represent ± SE. At week 3, letters indicate statistically significant differences among the four groups at P ≤ 0.05. No significant differences among groups were detected at other time points. N = 7.

The effect of the inoculation treatment on the time course of photosynthetic performance during drought as measured with F_V_/F_M_ differed between dry and wet families. During drought at week 3, F_V_/F_M_ of the inoculated group declined the fastest and was significantly lower than that of controls in the dry family (P < 0.05), but not the wet family (**Figure 3**). No statistically significant differences in F_V_/F_M_ existed between groups before drought or at other weeks (P > 0.05). Model selection parameters for F_V_/F_M_ are contained in **Supplementary Table 4**.

The taxonomic profile of the soil microbial community used for the inoculation treatment is presented in **Supplementary Tables 1** and **2**.

## Discussion

### Intraspecific Variation in Microbial Treatment Effects on Functional Traits

Consistent with our hypothesis, we found evidence that soil microbes can alter functional traits such as root:shoot biomass ratio that improve water and nutrient uptake and drought resistance more so in the wet family than in the dry family. The taxonomic profile of the soil microbial community used for the inoculation treatment contained drought-adapted bacteria (**Supplementary Table 5**), where the majority (60%) of the top ten most abundant fungal taxa was identified as Pleosporales in Ascomycota, which is dominant in arid soils (Porras-Alfaro et al., 2008; Porras-Alfaro et al., 2011). This supports its potential to benefit the wet family more than the dry family (Allsup and Lankau, 2019). The wet family’s significant and positive treatment effect size on root:shoot biomass ratio and the dry family nonsignificant effect size on root:shoot biomass ratio suggest that the inoculated group in the wet family allocated more resources belowground to increase root growth, which enhances plant water and nutrient acquisition, drought resistance, and the capacity to withstand drought (Brunner et al., 2015; Hagedorn et al., 2016). The inoculation treatment also differentially altered leaf %N, C:N, and δ^15^N between families where the effect sizes were opposite in sign between families.

Similar to root:shoot biomass ratio, F_V_/F_M_ was also differentially affected by the inoculation treatment between families. F_V_/F_M_ of the inoculated group declined faster than controls during the imposed drought only in the dry family and not the wet. One proposed mechanism underlying this observation is that the slightly greater height of the dry inoculated group compared to dry control group (albeit not significant) resulted in increased leaf area which may have contributed to faster desiccation and functional decline during drought. Another possible mechanism underlying the dry inoculated group’s F_V_/F_M_ decline may be related to the opposite (albeit not significant) effect sizes between families for %N and consequently C:N. In the wet family, we observed a positive treatment effect size on leaf %N and a negative effect size on C:N, while in contrast, the dry family exhibited the opposite: a negative effect size on leaf %N and a positive effect size on C:N. Increased C:N, as we observed in the dry inoculated group, can indicate greater drought stress (Chen et al., 2015; Ma et al., 2016). This may possibly be due to a negative interaction between the soil microbes and the dry family. (Enebak et al., 1998) also observed a negative effect of plant growth-promoting bacteria on loblolly pine seedling growth.

In addition to leaf %N and C:N, the family difference in inoculation treatment effect on F_V_/F_M_ may also be related to family differences in inoculation treatment effects on N uptake (e.g. NO_3_^-^, NH_4_^+^), as indicated by the moderately significant, positive treatment effect size on leaf δ^15^N in the dry family and the nonsignificant effect size in the wet family. Leaf δ^15^N reflects the ^15^N abundance of N sources available to plants (Shearer and Kohl, 1986). Our leaf δ^15^N results suggest that soil microbes may influence N uptake in the dry family differently than the wet family. This may occur because within-species families can interact differently with soil microbes by altering root exudate composition and quantity to recruit and repel different microbes (Haney et al., 2015). Because different soil microbes are responsible for transforming organic N to plant-accessible NH_4_^+^ or NO_3_^-^ (Hayatsu et al., 2008), the form of plant available N and thus leaf δ^15^N can differ between within-species families. The family differences in inoculation treatment effect sizes on leaf δ^15^N resulted in the dry inoculated group exhibiting the greatest leaf δ^15^N, which may indicate higher uptake of NH_4_^+^ (Miller and Bowman, 2002; Falkengren-Grerup et al., 2004), the primary form of N for loblolly (Lucash et al., 2008) and coniferous systems (Alexander, 1983). At NH_4_^+^ levels of 0.1 to 0.5 mmol/L, NH_4_^+^can be toxic to plants but the level at which it becomes toxic varies among species (Britto and Kronzucker, 2002). Therefore, it is possible that this could have contributed to the dry inoculated group’s decline in F_V_/F_M_ during drought at week 3 if the microbes in the inoculation treatment transformed N into relatively more NH_4_^+^ in the dry family compared to in the wet family. However, we present this only as a possibility because we cannot determine the quantity of NH_4_^+^ present given only the leaf δ^15^N data in this study. The low F_V_/F_M_ values of ~0.6 before drought suggest that seedlings may have been nutrient-deficient because they were grown in pure, fast-draining sand.

The taxonomic profile of the soil microbial community used for the inoculation treatment supports the possibility that the dry inoculated group’s F_V_/F_M_ decline may have been related to microbial effects on plant N availability due to the presence of fungi involved in plant N acquisition (**Supplementary Table 6**), which may have interacted with each seedling family differently. The majority of the top most abundant fungi was in Pleosporales, which contains dark septate fungi that can colonize *Pinus* species and are found in nutrient-stressed environments (Barrow, 2003), such as in our experiment using fast-draining sand. Dark septate fungi can form mutualistic relationships with plants by obtaining C from the plant in return for making nutrients available to the plant (Usuki and Narisawa, 2007; Wagg et al., 2008; Vergara et al., 2018), specifically transforming organic N into plant available forms (Alberton et al., 2010; Newsham, 2011; Bueno de Mesquita et al., 2018). The most abundant fungal genus in the soil microbial community used for the inoculation treatment was *Alternaria* of Pleosporales, which has been shown to vary with soil N availability (Porras-Alfaro et al., 2011). Given the presence of fungi involved in plant N acquisition, it is possible that the dry family’s significantly greater concentration of root exudates may have differentially affected the microbial community and resulted in different N types available compared to that of the wet family. This may have contributed to the observed variation in treatment effect size on leaf %N, C:N, and δ^15^N between families. Additionally, contrary to our hypothesis, the soil microbe treatment did not always influence functional traits that increased water and nutrient uptake and drought resistance (e.g. root:shoot biomass ratio) more in the wet family than in the dry family. This suggests that significant effects of soil microbes on plant function can vary depending on the trait of interest (Rincón et al., 2008). Regardless of family, inoculated plants exhibited significantly greater germination than controls, suggesting that soil microbes may improve seedling establishment regardless of family by directly or indirectly enhancing seed germination (Madhaiyan et al., 2005; Balshor et al., 2016). Soil microbes can directly enhance germination *via* the production of plant growth hormones (Wu et al., 2016), and also indirectly by increasing the soil water holding capacity of soil (Roberson and Firestone, 1992; Kaci et al., 2005; Or et al., 2007; Ulrich et al., 2019) and maintaining high soil moisture required for germination (Schultz, 1997), as well as increasing nutrient acquisition.

The taxonomic profile of the soil microbial community used for the inoculation treatment reflects its arid environment of origin and indicates the presence of fungi involved in plant N acquisition (**Supplementary Tables 5** and **6**). The majority (60%) of the top ten most abundant bacterial taxa was composed of Rubrobacterales in Actinobacteria, known to be thermophilic, radiation-resistant, and common in arid, desert soils (Holmes et al., 2000).

### Intraspecific Variation in Functional Traits Related to Seedling Drought Resistance and N Use

The dry family’s significantly greater root:shoot biomass ratio and root exudate concentration than that of the wet family suggests that the dry family may be more drought-adapted than the wet family. The dry family’s significantly greater root:shoot biomass ratio and concentration of root exudates supports its greater capacity to resist drought and reflects its drier climate of origin compared to the wet family (regardless of treatment). This greater belowground allocation of resources to root biomass enables the dry family to access deep water sources during limited water availability, a trait observed in families and populations adapted to drier climates (Brunner et al., 2015; Hagedorn et al., 2016). Greater allocation to root growth also enhances nutrient acquisition (López-Bucio et al., 2003; Hodge, 2004; Maire et al., 2009; Chapman et al., 2012), which can vary among loblolly pine families and populations (Li et al., 1991). The dry family’s greater allocation to belowground growth may also underlie why we observed a significant treatment effect size on root:shoot biomass in only the wet family, as the dry family was already allocating more resources belowground independent of treatment. The dry family was also significantly shorter than the wet family, reducing leaf area through which water can be lost, another mechanism of drought resistance.

The dry family’s significantly greater concentration of root exudates compared to the wet family may be used to shape the soil microbial community by attracting or repelling different microbes (Bever et al., 2012; Sasse et al., 2018; Zhalnina et al., 2018), another strategy to increase water and nutrient uptake, enhance drought resistance (Badri and Vivanco, 2009; Lareen et al., 2016; Jacoby et al., 2017), and alter the water holding capacity of the soil (Young, 1995; Angers and Caron, 1998; Walker et al., 2003). Families adapted to drier or wetter climates can differ in their root exudation quantity and quality (Whitham et al., 2003) because root exudate quantity can increase during drought (Liese et al., 2018; Preece et al., 2018) and root exudate composition can also change as a result of dry conditions (Gargallo-Garriga et al., 2018). Furthermore, root exudate rates and composition have been shown to vary across genotypes (Cieslinski et al., 1997; Hoekenga et al., 2003; Badri and Vivanco, 2009) and influence the genotype-specific ability to recruit beneficial soil microbes (Haney et al., 2015).

The dry and wet families may have used different sources of plant available N (e.g. NO_3_^-^, NH_4_^+^), as suggested by the dry family’s significantly greater leaf δ^15^N than the wet family regardless of treatment. N use is driven by the quantity of N forms available in the soil and the capacity of plants to take up different N forms (Vasquez et al., 2008). Within-species pine populations and families can differ in their use of NH_4_^+^ or NO_3_^-^ (Miller and Hawkins, 2007; Maire et al., 2009) because plants can switch their N source (Louahlia et al., 2000) depending on precipitation (Houlton et al., 2007) and season (Lucash et al., 2008), suggesting that plasticity exists in the uptake of different N sources between within-species populations and families from contrasting climates. Plasticity in N use can be driven by within-species variation in root morphology, preferential expression of N transporters (NO_3_^-^ versus NH_4_^+^) (Maire et al., 2009), and/or activity patterns of soil enzymes involved in the acquisition of N (Purahong et al., 2016). However, it remains unclear if the relationship between shoot δ^15^N and N source is universal (Kahmen et al., 2008; Temperton et al., 2012) because the δ^15^N of NO_3_^-^ and NH_4_^+^ can vary substantially depending on relative mineralization, nitrification, and denitrification (Shearer et al., 1974; Mariotti et al., 1981; Handley et al., 1999).

The family differences in N use may be related to the family differences in root exudates because the dry family both released greater root exudates and exhibited greater leaf δ^15^N than the wet family. Root exudates can alter the availability of plant available N sources by stimulating microbial growth and activity (Marschner, 2012). An increase in microbial activity can increase microbial N transformations and influence the quantity and type of plant available N (Yin et al., 2013). Root exudates may also affect the N cycling function of the soil microbial community by attracting or repelling specific microbes that may promote the plant availability of NO_3_^-^ or NH_4_^+^ and alter the NO_3_^-^:NH_4_^+^ ratio (Hayatsu et al., 2008). Root exudates can also alter N availability by influencing different steps of the N cycle such as inhibiting nitrification (Subbarao et al., 2006; Coskun et al., 2017).

Despite significant family differences in root:shoot biomass, root exudates, and δ^15^N, we did not observe significant family or treatment differences in functional traits related to leaf drought tolerance, intrinsic water use efficiency, and stomatal constraints on gas exchange as indicated by Ψ_TLP_, δ^13^C, *A*, and *g*_s_, respectively. This suggests that Ψ_TLP_, a metric of drought tolerance (Bartlett et al., 2014), can reflect the conditions under which plant tissues develop and can outweigh ecotypic differences between families and populations. Meier et al. (1992) also did not find significant differences in Ψ_TLP_ between *P. taeda* families and populations from contrasting climates. For leaf δ^13^C, a metric of intrinsic water use efficiency (Farquhar et al., 1989), some studies of conifer species have observed intraspecific variation in leaf δ^13^C reflecting climate of origin (Yang et al., 2002; Kerr et al., 2015; Marias et al., 2017) while others have not (Zhang et al., 1993; Zhang et al., 1997). For *A*, and *g*_s_, others also have not observed significant differences in gas exchange parameters among within-species loblolly pine families and populations (Yang et al., 2002; Aspinwall et al., 2011). Given these mixed results, future provenance studies have been urged to focus on growth and survival traits rather than δ^13^C as a proxy for drought resistance (Moran et al., 2017).

## Conclusion

Our results showed that soil microbial inoculation treatment effects varied in direction and magnitude by trait and family. Soil microbes may alter functional traits that improve water and nutrient uptake and drought resistance such as the root:shoot biomass ratio more so in a family originating from a wetter climate than in a family originating from a drier climate. Regardless of treatment, the dry family exhibited a greater root:shoot biomass ratio, root exudate concentration, and leaf δ^15^N than the wet family. This suggests that the dry family allocated more resources belowground than the wet, and that within-species families may have used different sources of plant available N, which may be related to their climate and soil of origin. Together, this work highlights the need to further investigate in diverse systems how abiotic factors like drought and biotic factors like soil microbes influence diverse functional traits that influence seedling establishment of families and populations from contrasting climates. Given the mix of positive and negative microbial treatment effects on the dry family (e.g. increased germination but reduced F_V_/F_M_), more research is needed to inform plant-microbe interactions by identifying potential physiological tradeoffs due to negative interactions with soil microbes. Examination of intraspecific variation in plant physiological impacts of soil microbes informs species’ distributions, improves efforts to engineer beneficial plant-microbe interactions, and facilitates seedling adaptation and reforestation under future climate regimes.

## Data Availability Statement

The datasets generated for this study can be found in the Supplementary Information of this article.

## Author Contributions

DU, SS, and JD conceived and designed the study. DU, MR, and SP set up the experiment and made all measurements. DU conducted data analysis and wrote the first draft of the manuscript. All authors revised and approved of the final version of the manuscript.

## Funding

This research was funded by the Los Alamos National Laboratory Climate Space and Earth Science Chick Keller Postdoctoral Fellowship, the Los Alamos National Laboratory Directed Research and Development project #20160373ER, and the U.S. Department of Energy Biological System Science Division Science Focus Area Grant (2015SFAF260 and 2019SFAF255).

## Conflict of Interest

The authors declare that the research was conducted in the absence of any commercial or financial relationships that could be construed as a potential conflict of interest.
