# Supplementary material for: Effects of Soil Microbes on Functional Traits of Loblolly Pine (Pinus taeda) Seedling Families From Contrasting Climates
Source: Front Plant Sci. 2020 Jan 9;10:1643. doi: 10.3389/fpls.2019.01643 (PMC6962191; doi:10.3389/fpls.2019.01643)
Supplement: Supplementary file 1 [file DataSheet_1.docx]

**SUPPLEMENTARY INFORMATION**

**Supplementary Table 1.** Effect size mean and 95% confidence intervals (lower, upper) of treatment on traits of dry and wet families. Effect size was considered to be significant if the 95% confidence interval did not overlap with an effect size of 0. Effect size was considered to be moderately significant if the 95% confidence interval overlapped an effect size of 0 by less than ±0.1. A positive effect size indicates an increase in the functional trait.

|  | **Trait** | **Effect size mean** | **Lower** | **Upper** |
| --- | --- | --- | --- | --- |
| **Dry** | Germination | 1.74 | 0.11 | 3.36 |
|  | Height | 0.53 | -0.88 | 1.94 |
|  | Root:Shoot | -0.16 | -1.55 | 1.23 |
|  | Root exudates | -0.04 | -1.43 | 1.35 |
|  | d15N | 1.53 | -0.04 | 3.11 |
|  | %C | -0.41 | -1.82 | 0.99 |
|  | %N | -0.93 | -2.38 | 0.53 |
|  | C:N | 1.08 | -0.40 | 2.56 |
|  | d13C | -0.48 | -1.88 | 0.93 |
|  | A | -0.79 | -2.22 | 0.65 |
|  | gs | -0.99 | -2.46 | 0.47 |
|  | TLP | 0.17 | -1.21 | 1.56 |
|  | | | | |
| **Wet** | Germination | 1.74 | 0.11 | 3.36 |
|  | Height | 0.00 | -1.39 | 1.39 |
|  | Root:Shoot | 1.66 | 0.05 | 3.27 |
|  | Root exudates | -0.27 | -1.66 | 1.13 |
|  | d15N | 0.02 | -1.37 | 1.41 |
|  | %C | -0.11 | -1.50 | 1.28 |
|  | %N | 1.17 | -0.33 | 2.67 |
|  | C:N | -1.32 | -2.84 | 0.21 |
|  | d13C | -0.56 | -1.97 | 0.86 |
|  | A | -0.78 | -2.22 | 0.66 |
|  | gs | -0.92 | -2.38 | 0.53 |
|  | TLP | 0.07 | -1.31 | 1.46 |

**Supplementary Table 2.** ANOVA tables for effects of treatment, family, and treatment*family on functional traits: germination, height, root:shoot biomass ratio, root exudate concentration (conc), leaf nitrogen isotope ratios (δ^15^N), leaf C content (%C), leaf N content (%N), carbon:nitrogen ratio (C:N), leaf carbon isotope ratio (δ^13^C), photosynthesis (*A*), stomatal conductance (*g*_s_), and turgor loss point (TLP). Degrees of freedom (Df), sum of squares (Sum Sq), mean squares (Mean Sq).

| **Trait** | **Effect** | **Df** | **Sum Sq** | **Mean Sq** | **F-value** | **P-value** |
| --- | --- | --- | --- | --- | --- | --- |
| Germination | Treatment | 1 | 16 | 16 | 3.47E+31 | <2E-16 |
|  | Family | 1 | 0 | 0 | 2.17E+00 | 0.167 |
|  | Treatment x Family | 1 | 0 | 0 | 7.23E-01 | 0.412 |
|  | Residuals | 12 | 0 | 0 |  |  |
|  |  |  |  |  |  |  |
| Height | Treatment | 1 | 0.031 | 0.0310 | 0.0260 | 0.874 |
|  | Family | 1 | 10.1 | 10.1 | 8.65 | 0.0123 |
|  | Treatment x Family | 1 | 5.88 | 5.88 | 5.05 | 0.0442 |
|  | Residuals | 12 | 13.9 | 1.17 |  |  |
|  |  |  |  |  |  |  |
| Root:shoot biomass | Treatment | 1 | 0.0550 | 0.0551 | 1.545 | 0.780 |
|  | Family | 1 | 0.363 | 0.363 | 10.19 | 0.00179 |
|  | Treatment x Family | 1 | 0.0640 | 0.0640 | 1.796 | 0.120 |
|  | Residuals | 11 | 4.42 | 0.0356 |  |  |
|  |  |  |  |  |  |  |
| Root exudate conc | Treatment | 1 | 2.14 | 2.14 | 0.102 | 0.755 |
|  | Family | 1 | 99.8 | 99.82 | 4.772 | 0.0495 |
|  | Treatment x Family | 1 | 0.990 | 0.99 | 0.047 | 0.832 |
|  | Residuals | 12 | 251.0 | 20.92 |  |  |
|  |  |  |  |  |  |  |
| Leaf δ^15^N | Treatment | 1 | 0.825 | 0.825 | 1.181 | 0.300 |
|  | Family | 1 | 16.5 | 16.456 | 23.6 | 5.08E-4 |
|  | Treatment x Family | 1 | 1.12 | 1.124 | 1.609 | 0.231 |
|  | Residuals | 11 | 7.69 | 0.699 |  |  |
|  |  |  |  |  |  |  |
| %C | Treatment | 1 | 0.129 | 0.130 | 0.244 | 0.631 |
|  | Family | 1 | 0.817 | 0.817 | 1.538 | 0.241 |
|  | Treatment x Family | 1 | 0.045 | 0.0453 | 0.085 | 0.776 |
|  | Residuals | 11 | 5.84 | 0.531 |  |  |
|  |  |  |  |  |  |  |
| %N | Treatment | 1 | 1.35E-3 | 0.00135 | 0.235 | 0.637 |
|  | Family | 1 | 9.1E-4 | 9.1E-4 | 0.159 | 0.698 |
|  | Treatment x Family | 1 | 0.0252 | 0.0252 | 4.385 | 0.0602 |
|  | Residuals | 11 | 0.0633 | 0.00575 |  |  |
|  |  |  |  |  |  |  |
| C:N | Treatment | 1 | 14.2 | 14.2 | 0.088 | 0.772 |
|  | Family | 1 | 48.9 | 48.9 | 0.304 | 0.593 |
|  | Treatment x Family | 1 | 1002.0 | 1002.0 | 6.22 | 0.0298 |
|  | Residuals | 11 | 1771.4 | 161 |  |  |
|  |  |  |  |  |  |  |
| Leaf δ^13^C | Treatment | 1 | 1.54 | 1.54 | 1.187 | 0.299 |
|  | Family | 1 | 9.0E-3 | 8.8E-3 | 0.007 | 0.936 |
|  | Treatment x Family | 1 | 1.0E-3 | 9.1E-4 | 0.001 | 0.979 |
|  | Residuals | 11 | 14.3 | 1.30 |  |  |
|  |  |  |  |  |  |  |
| *A* | Treatment | 1 | 14.6 | 14.6 | 3.167 | 0.1 |
|  | Family | 1 | 0.600 | 0.602 | 0.131 | 0.724 |
|  | Treatment x Family | 1 | 0.390 | 0.389 | 0.084 | 0.776 |
|  | Residuals | 12 | 55.3 | 4.61 |  |  |
|  |  |  |  |  |  |  |
| *g*_s_ | Treatment | 1 | 4.58E-3 | 4.58E-3 | 4.848 | 0.048 |
|  | Family | 1 | 3.0E-6 | 3.0E-6 | 4.0E-3 | 0.953 |
|  | Treatment x Family | 1 | 1.18E-4 | 1.18E-4 | 0.125 | 0.729 |
|  | Residuals | 12 | 1.13E-2 | 9.44E-4 |  |  |
|  |  |  |  |  |  |  |
| TLP | Treatment | 1 | 0.290 | 0.29 | 0.184 | 0.677 |
|  | Family | 1 | 3.79 | 3.79 | 2.41 | 0.152 |
|  | Treatment x Family | 1 | 0 | 0 | 0 | 0.995 |
|  | Residuals | 10 | 15.7 | 1.573 |  |  |

**Supplementary Table 3.** ANOVA tables for effects of treatment, family, and treatment*family on pressure-volume (PV) curve parameters: water potential at the turgor loss point (Ψ_TLP_), the water potential at full turgor (Ψ_100_), the modulus of elasticity (ε), apoplastic water fraction (Apoplastic), symplastic water fraction (Symplastic), and relative water content at the turgor loss point (RWC@TLP). Degrees of freedom (Df), sum of squares (Sum Sq), mean squares (Mean Sq).

| **Parameter** | **Effect** | **Df** | **Sum Sq** | **Mean Sq** | **F-value** | **P-value** |
| --- | --- | --- | --- | --- | --- | --- |
| **Ψ_TLP_** | Treatment | 1 | 0.29 | 0.29 | 0.184 | 0.677 |
|  | Family | 1 | 3.793 | 3.793 | 2.411 | 0.152 |
|  | Treatment x Family | 1 | 0 | 0 | 0 | 0.995 |
|  | Residuals | 10 | 15.732 | 1.573 |  |  |
|  |  |  |  |  |  |  |
| **Ψ_100_** | Treatment | 1 | 0.49 | 0.4899 | 0.764 | 0.402 |
|  | Family | 1 | 0.609 | 0.6088 | 0.95 | 0.353 |
|  | Treatment x Family | 1 | 0.026 | 0.0256 | 0.04 | 0.846 |
|  | Residuals | 10 | 6.409 | 0.6409 |  |  |
|  |  |  |  |  |  |  |
| **ε** | Treatment | 1 | 712432 | 712432 | 0.215 | 0.653 |
|  | Family | 1 | 1657129 | 1657129 | 0.5 | 0.496 |
|  | Treatment x Family | 1 | 140611 | 140611 | 0.042 | 0.841 |
|  | Residuals | 10 | 33127216 | 3312722 |  |  |
|  |  |  |  |  |  |  |
| **Apoplastic** | Treatment | 1 | 0.377 | 0.3767 | 0.211 | 0.655 |
|  | Family | 1 | 0.781 | 0.7812 | 0.438 | 0.523 |
|  | Treatment x Family | 1 | 0.186 | 0.1858 | 0.104 | 0.753 |
|  | Residuals | 10 | 17.817 | 1.7817 |  |  |
|  |  |  |  |  |  |  |
| **Symplastic** | Treatment | 1 | 0.377 | 0.3767 | 0.211 | 0.655 |
|  | Family | 1 | 0.781 | 0.7812 | 0.438 | 0.523 |
|  | Treatment x Family | 1 | 0.186 | 0.1858 | 0.104 | 0.753 |
|  | Residuals | 10 | 17.817 | 1.7817 |  |  |
|  |  |  |  |  |  |  |
| **RWC@TLP** | Treatment | 1 | 0.004 | 0.0037 | 0.004 | 0.952 |
|  | Family | 1 | 0.03 | 0.03 | 0.032 | 0.862 |
|  | Treatment x Family | 1 | 1.189 | 1.1891 | 1.257 | 0.288 |
|  | Residuals | 10 | 9.458 | 0.9458 |  |  |

**Supplementary Table 4.** Model selection parameters for F_V_/F_M_ timecourses. Degrees of freedom (Df), Akaike Information Criterion (AIC), Bayesian Information Criterion (BIC).

| **Model** | **Df** | **AIC** | **BIC** | **Log-likelihood** |
| --- | --- | --- | --- | --- |
| General correlation structure | 22 | -1032.333 | -917.8584 | 538.1667 |
| Compound symmetry | 22 | -1033.434 | -918.9595 | 538.7172 |

**Supplementary Table 5.** Bacterial taxonomy in the soil microbial community used for the inoculation treatment. Taxa are ordered by count and then alphabetically by phyla, order, family, and genus.

| **Phyla** | **Order** | **Family** | **Genus** | **Count** |
| --- | --- | --- | --- | --- |
| Actinobacteria | Rubrobacterales | Rubrobacteraceae | Rubrobacter | 355 |
| Actinobacteria | Rubrobacterales | Rubrobacteraceae | Rubrobacter | 101 |
| Actinobacteria | Rubrobacterales | Rubrobacteraceae | Rubrobacter | 94 |
| Actinobacteria | Rubrobacterales | Rubrobacteraceae | Rubrobacter | 81 |
| Acidobacteria | Gp6 | Gp6 | Gp6 | 60 |
| Acidobacteria | Gp4 | Gp4 | Gp4 | 58 |
| Actinobacteria | Rubrobacterales | Rubrobacteraceae | Rubrobacter | 34 |
| Bacteroidetes | Sphingobacteriales | Chitinophagaceae | Segetibacter | 31 |
| Proteobacteria | Rhizobiales | Methylobacteriaceae | Microvirga | 31 |
| Actinobacteria | Rubrobacterales | Rubrobacteraceae | Rubrobacter | 29 |
| Actinobacteria | Rubrobacterales | Rubrobacteraceae | Rubrobacter | 26 |
| Acidobacteria | Gp4 | Gp4 | Gp4 | 21 |
| Actinobacteria | Solirubrobacterales | Conexibacteraceae | Conexibacter | 20 |
| Actinobacteria | Solirubrobacterales | Solirubrobacteraceae | Solirubrobacter | 20 |
| Acidobacteria | Gp16 | Gp16 | Gp16 | 14 |
| Actinobacteria | Gaiellales | Gaiellaceae | Gaiella | 14 |
| Actinobacteria | Actinomycetales | Geodermatophilaceae | Blastococcus | 13 |
| Actinobacteria | Actinomycetales | Geodermatophilaceae | Geodermatophilus | 13 |
| Armatimonadetes | Armatimonadales | Armatimonadaceae | Armatimonas/Armatimonadetes_gp1 | 13 |
| Acidobacteria | Gp4 | Gp4 | Gp4 | 12 |
| Actinobacteria | Actinomycetales | Geodermatophilaceae | Modestobacter | 11 |
| Acidobacteria | Gp3 | Gp3 | Gp3 | 9 |
| Acidobacteria | Gp4 | Gp4 | Gp4 | 9 |
| Acidobacteria | Gp4 | Gp4 | Gp4 | 9 |
| Actinobacteria | Rubrobacterales | Rubrobacteraceae | Rubrobacter | 9 |
| Actinobacteria | Rubrobacterales | Rubrobacteraceae | Rubrobacter | 9 |
| Actinobacteria | Actinomycetales | Geodermatophilaceae | Geodermatophilus | 8 |
| Verrucomicrobia | Spartobacteria_genera_incertae_sedis | Spartobacteria_genera_incertae_sedis | Spartobacteria_genera_incertae_sedis | 8 |
| Acidobacteria | Gp6 | Gp6 | Gp6 | 7 |
| Acidobacteria | Gp7 | Gp7 | Gp7 | 7 |
| Actinobacteria | Actinomycetales | Micrococcaceae | Arthrobacter | 7 |
| Actinobacteria | Actinomycetales | Micromonosporaceae | Asanoa | 7 |
| Acidobacteria | Gp4 | Gp4 | Gp4 | 6 |
| Actinobacteria | Gaiellales | Gaiellaceae | Gaiella | 6 |
| Actinobacteria | Rubrobacterales | Rubrobacteraceae | Rubrobacter | 6 |
| Bacteroidetes | Sphingobacteriales | Chitinophagaceae | Segetibacter | 6 |
| candidate division WPS-1 | WPS-1_genera_incertae_sedis | WPS-1_genera_incertae_sedis | WPS-1_genera_incertae_sedis | 6 |
| Nitrospirae | Nitrospirales | Nitrospiraceae | Nitrospira | 6 |
| Verrucomicrobia | Spartobacteria_genera_incertae_sedis | Spartobacteria_genera_incertae_sedis | Spartobacteria_genera_incertae_sedis | 6 |
| Actinobacteria | Actinomycetales | Pseudonocardiaceae | Pseudonocardia | 5 |
| Actinobacteria | Gaiellales | Gaiellaceae | Gaiella | 5 |
| Actinobacteria | Gaiellales | Gaiellaceae | Gaiella | 5 |
| Actinobacteria | Solirubrobacterales | Conexibacteraceae | Conexibacter | 5 |
| Bacteroidetes | Sphingobacteriales | Chitinophagaceae | Flavisolibacter | 5 |
| Bacteroidetes | Sphingobacteriales | Chitinophagaceae | Segetibacter | 5 |
| Gemmatimonadetes | Gemmatimonadales | Gemmatimonadaceae | Gemmatimonas | 5 |
| Nitrospirae | Nitrospirales | Nitrospiraceae | Nitrospira | 5 |
| Acidobacteria | Gp16 | Gp16 | Gp16 | 4 |
| Actinobacteria | Actinomycetales | Nocardioidaceae | Nocardioides | 4 |
| Actinobacteria | Gaiellales | Gaiellaceae | Gaiella | 4 |
| Bacteroidetes | Sphingobacteriales | Chitinophagaceae | Flavisolibacter | 4 |
| candidate division WPS-1 | WPS-1_genera_incertae_sedis | WPS-1_genera_incertae_sedis | WPS-1_genera_incertae_sedis | 4 |
| Chloroflexi | Sphaerobacterales | Sphaerobacteraceae | Sphaerobacter | 4 |
| Gemmatimonadetes | Gemmatimonadales | Gemmatimonadaceae | Gemmatimonas | 4 |
| Proteobacteria | Rhodospirillales | Acetobacteraceae | Belnapia | 4 |
| Acidobacteria | Blastocatella | Blastocatella | Blastocatella | 3 |
| Acidobacteria | Gp16 | Gp16 | Gp16 | 3 |
| Acidobacteria | Gp4 | Gp4 | Gp4 | 3 |
| Acidobacteria | Gp6 | Gp6 | Gp6 | 3 |
| Acidobacteria | Gp6 | Gp6 | Gp6 | 3 |
| Acidobacteria | Gp6 | Gp6 | Gp6 | 3 |
| Actinobacteria | Actinomycetales | Kineosporiaceae | Kineosporia | 3 |
| Actinobacteria | Actinomycetales | Nocardioidaceae | Kribbella | 3 |
| Actinobacteria | Actinomycetales | Nocardioidaceae | Nocardioides | 3 |
| Actinobacteria | Gaiellales | Gaiellaceae | Gaiella | 3 |
| Actinobacteria | Gaiellales | Gaiellaceae | Gaiella | 3 |
| Actinobacteria | Gaiellales | Gaiellaceae | Gaiella | 3 |
| Actinobacteria | Gaiellales | Gaiellaceae | Gaiella | 3 |
| Actinobacteria | Gaiellales | Gaiellaceae | Gaiella | 3 |
| Actinobacteria | Thermoleophilales | Thermoleophilaceae | Thermoleophilum | 3 |
| Bacteroidetes | Cytophagales | Cytophagaceae | Adhaeribacter | 3 |
| Bacteroidetes | Sphingobacteriales | Chitinophagaceae | Segetibacter | 3 |
| candidate division WPS-1 | WPS-1_genera_incertae_sedis | WPS-1_genera_incertae_sedis | WPS-1_genera_incertae_sedis | 3 |
| Gemmatimonadetes | Gemmatimonadales | Gemmatimonadaceae | Gemmatimonas | 3 |
| Proteobacteria | Sphingomonadales | Sphingomonadaceae | Sphingomonas | 3 |
| Acidobacteria | Gp3 | Gp3 | Gp3 | 2 |
| Acidobacteria | Gp3 | Gp3 | Gp3 | 2 |
| Acidobacteria | Gp4 | Gp4 | Gp4 | 2 |
| Acidobacteria | Gp4 | Gp4 | Gp4 | 2 |
| Acidobacteria | Gp6 | Gp6 | Gp6 | 2 |
| Actinobacteria | Actinomycetales | Geodermatophilaceae | Blastococcus | 2 |
| Actinobacteria | Actinomycetales | Geodermatophilaceae | Blastococcus | 2 |
| Actinobacteria | Actinomycetales | Geodermatophilaceae | Modestobacter | 2 |
| Actinobacteria | Actinomycetales | Nocardioidaceae | Marmoricola | 2 |
| Actinobacteria | Actinomycetales | Nocardioidaceae | Marmoricola | 2 |
| Actinobacteria | Actinomycetales | Nocardioidaceae | Nocardioides | 2 |
| Actinobacteria | Actinomycetales | Propionibacteriaceae | Microlunatus | 2 |
| Actinobacteria | Gaiellales | Gaiellaceae | Gaiella | 2 |
| Actinobacteria | Gaiellales | Gaiellaceae | Gaiella | 2 |
| Actinobacteria | Gaiellales | Gaiellaceae | Gaiella | 2 |
| Actinobacteria | Gaiellales | Gaiellaceae | Gaiella | 2 |
| Actinobacteria | Gaiellales | Gaiellaceae | Gaiella | 2 |
| Armatimonadetes | Armatimonadales | Armatimonadaceae | Armatimonas/Armatimonadetes_gp1 | 2 |
| Armatimonadetes | Armatimonadales | Armatimonadaceae | Armatimonas/Armatimonadetes_gp1 | 2 |
| Bacteroidetes | Sphingobacteriales | Chitinophagaceae | Flavisolibacter | 2 |
| Bacteroidetes | Sphingobacteriales | Chitinophagaceae | Flavitalea | 2 |
| Deinococcus-Thermus | Deinococcales | Trueperaceae | Truepera | 2 |
| Firmicutes | Bacillales | Planococcaceae | Sporosarcina | 2 |
| Gemmatimonadetes | Gemmatimonadales | Gemmatimonadaceae | Gemmatimonas | 2 |
| Gemmatimonadetes | Gemmatimonadales | Gemmatimonadaceae | Gemmatimonas | 2 |
| Proteobacteria | Ferritrophicales | Ferritrophicaceae | Ferritrophicum | 2 |
| Proteobacteria | Oceanospirillales | Halomonadaceae | Halomonas | 2 |
| Proteobacteria | Rhizobiales | Beijerinckiaceae | Chelatococcus | 2 |
| Proteobacteria | Rhizobiales | Rhizobiaceae | Rhizobium | 2 |
| Proteobacteria | Rhodospirillales | Acetobacteraceae | Belnapia | 2 |
| Proteobacteria | Sphingomonadales | Sphingomonadaceae | Sphingomonas | 2 |
| Proteobacteria | Sphingomonadales | Sphingomonadaceae | Sphingomonas | 2 |
| Proteobacteria | Sphingomonadales | Sphingomonadaceae | Sphingomonas | 2 |
| Proteobacteria | Sphingomonadales | Sphingomonadaceae | Sphingomonas | 2 |
| Acidobacteria | Aridibacter | Aridibacter | Aridibacter | 1 |
| Acidobacteria | Aridibacter | Aridibacter | Aridibacter | 1 |
| Acidobacteria | Aridibacter | Aridibacter | Aridibacter | 1 |
| Acidobacteria | Aridibacter | Aridibacter | Aridibacter | 1 |
| Acidobacteria | Gp10 | Gp10 | Gp10 | 1 |
| Acidobacteria | Gp3 | Gp3 | Gp3 | 1 |
| Acidobacteria | Gp3 | Gp3 | Gp3 | 1 |
| Acidobacteria | Gp7 | Gp7 | Gp7 | 1 |
| Acidobacteria | Gp7 | Gp7 | Gp7 | 1 |
| Actinobacteria | Acidimicrobiales | Acidimicrobineae_incertae_sedis | Aciditerrimonas | 1 |
| Actinobacteria | Acidimicrobiales | Acidimicrobineae_incertae_sedis | Aciditerrimonas | 1 |
| Actinobacteria | Acidimicrobiales | Acidimicrobineae_incertae_sedis | Aciditerrimonas | 1 |
| Actinobacteria | Acidimicrobiales | Acidimicrobineae_incertae_sedis | Aciditerrimonas | 1 |
| Actinobacteria | Actinomycetales | Cellulomonadaceae | Cellulomonas | 1 |
| Actinobacteria | Actinomycetales | Cellulomonadaceae | Cellulomonas | 1 |
| Actinobacteria | Actinomycetales | Cellulomonadaceae | Cellulomonas | 1 |
| Actinobacteria | Actinomycetales | Geodermatophilaceae | Geodermatophilus | 1 |
| Actinobacteria | Actinomycetales | Microbacteriaceae | Curtobacterium | 1 |
| Actinobacteria | Actinomycetales | Micromonosporaceae | Actinoplanes | 1 |
| Actinobacteria | Actinomycetales | Micromonosporaceae | Actinoplanes | 1 |
| Actinobacteria | Actinomycetales | Micromonosporaceae | Virgisporangium | 1 |
| Actinobacteria | Actinomycetales | Nocardioidaceae | Aeromicrobium | 1 |
| Actinobacteria | Actinomycetales | Nocardioidaceae | Nocardioides | 1 |
| Actinobacteria | Actinomycetales | Pseudonocardiaceae | Actinomycetospora | 1 |
| Actinobacteria | Actinomycetales | Pseudonocardiaceae | Pseudonocardia | 1 |
| Actinobacteria | Actinomycetales | Streptomycetaceae | Streptomyces | 1 |
| Actinobacteria | Actinomycetales | Streptosporangiaceae | Streptosporangium | 1 |
| Actinobacteria | Actinomycetales | Thermomonosporaceae | Actinomadura | 1 |
| Actinobacteria | Gaiellales | Gaiellaceae | Gaiella | 1 |
| Actinobacteria | Gaiellales | Gaiellaceae | Gaiella | 1 |
| Actinobacteria | Gaiellales | Gaiellaceae | Gaiella | 1 |
| Actinobacteria | Gaiellales | Gaiellaceae | Gaiella | 1 |
| Actinobacteria | Gaiellales | Gaiellaceae | Gaiella | 1 |
| Actinobacteria | Gaiellales | Gaiellaceae | Gaiella | 1 |
| Actinobacteria | Gaiellales | Gaiellaceae | Gaiella | 1 |
| Actinobacteria | Gaiellales | Gaiellaceae | Gaiella | 1 |
| Actinobacteria | Gaiellales | Gaiellaceae | Gaiella | 1 |
| Actinobacteria | Gaiellales | Gaiellaceae | Gaiella | 1 |
| Actinobacteria | Gaiellales | Gaiellaceae | Gaiella | 1 |
| Actinobacteria | Solirubrobacterales | Conexibacteraceae | Conexibacter | 1 |
| Actinobacteria | Solirubrobacterales | Conexibacteraceae | Conexibacter | 1 |
| Actinobacteria | Solirubrobacterales | Solirubrobacteraceae | Solirubrobacter | 1 |
| Armatimonadetes | Armatimonadales | Armatimonadaceae | Armatimonas/Armatimonadetes_gp1 | 1 |
| Armatimonadetes | Armatimonadetes_gp4 | Armatimonadetes_gp4 | Armatimonadetes_gp4 | 1 |
| Armatimonadetes | Armatimonadetes_gp4 | Armatimonadetes_gp4 | Armatimonadetes_gp4 | 1 |
| Bacteroidetes | Cytophagales | Cytophagaceae | Adhaeribacter | 1 |
| Bacteroidetes | Cytophagales | Cytophagaceae | Adhaeribacter | 1 |
| Bacteroidetes | Cytophagales | Cytophagaceae | Adhaeribacter | 1 |
| Bacteroidetes | Cytophagales | Cytophagaceae | Hymenobacter | 1 |
| Bacteroidetes | Cytophagales | Cytophagaceae | Hymenobacter | 1 |
| Bacteroidetes | Cytophagales | Cytophagaceae | Rhodocytophaga | 1 |
| Bacteroidetes | Cytophagales | Cytophagaceae | Rhodocytophaga | 1 |
| Bacteroidetes | Cytophagales | Cytophagaceae | Rhodocytophaga | 1 |
| Bacteroidetes | Cytophagales | Cytophagaceae | Spirosoma | 1 |
| Bacteroidetes | Flavobacteriales | Flavobacteriaceae | Flavobacterium | 1 |
| Bacteroidetes | Sphingobacteriales | Chitinophagaceae | Ferruginibacter | 1 |
| Bacteroidetes | Sphingobacteriales | Chitinophagaceae | Flavisolibacter | 1 |
| Bacteroidetes | Sphingobacteriales | Chitinophagaceae | Flavisolibacter | 1 |
| Bacteroidetes | Sphingobacteriales | Chitinophagaceae | Flavisolibacter | 1 |
| Bacteroidetes | Sphingobacteriales | Chitinophagaceae | Flavisolibacter | 1 |
| Bacteroidetes | Sphingobacteriales | Chitinophagaceae | Segetibacter | 1 |
| Bacteroidetes | Sphingobacteriales | Chitinophagaceae | Segetibacter | 1 |
| Bacteroidetes | Sphingobacteriales | Chitinophagaceae | Segetibacter | 1 |
| Bacteroidetes | Sphingobacteriales | Sphingobacteriaceae | Pedobacter | 1 |
| candidate division WPS-1 | WPS-1_genera_incertae_sedis | WPS-1_genera_incertae_sedis | WPS-1_genera_incertae_sedis | 1 |
| candidate division WPS-1 | WPS-1_genera_incertae_sedis | WPS-1_genera_incertae_sedis | WPS-1_genera_incertae_sedis | 1 |
| candidate division WPS-1 | WPS-1_genera_incertae_sedis | WPS-1_genera_incertae_sedis | WPS-1_genera_incertae_sedis | 1 |
| Chloroflexi | Caldilineales | Caldilineaceae | Litorilinea | 1 |
| Chloroflexi | Caldilineales | Caldilineaceae | Litorilinea | 1 |
| Chloroflexi | Caldilineales | Caldilineaceae | Litorilinea | 1 |
| Chloroflexi | Caldilineales | Caldilineaceae | Litorilinea | 1 |
| Chloroflexi | Kallotenuales | Kallotenuaceae | Kallotenue | 1 |
| Chloroflexi | Kallotenuales | Kallotenuaceae | Kallotenue | 1 |
| Chloroflexi | Kallotenuales | Kallotenuaceae | Kallotenue | 1 |
| Cyanobacteria/Chloroplast | Family XIII | Family XIII | GpXIII | 1 |
| Cyanobacteria/Chloroplast | Family XIII | Family XIII | GpXIII | 1 |
| Deinococcus-Thermus | Deinococcales | Trueperaceae | Truepera | 1 |
| Firmicutes | Bacillales | Paenibacillaceae 1 | Paenibacillus | 1 |
| Firmicutes | Bacillales | Thermoactinomycetaceae 1 | Shimazuella | 1 |
| Gemmatimonadetes | Gemmatimonadales | Gemmatimonadaceae | Gemmatimonas | 1 |
| Gemmatimonadetes | Gemmatimonadales | Gemmatimonadaceae | Gemmatimonas | 1 |
| Gemmatimonadetes | Gemmatimonadales | Gemmatimonadaceae | Gemmatimonas | 1 |
| Gemmatimonadetes | Gemmatimonadales | Gemmatimonadaceae | Gemmatimonas | 1 |
| Gemmatimonadetes | Gemmatimonadales | Gemmatimonadaceae | Gemmatimonas | 1 |
| Planctomycetes | Planctomycetales | Planctomycetaceae | Aquisphaera | 1 |
| Planctomycetes | Planctomycetales | Planctomycetaceae | Gemmata | 1 |
| Planctomycetes | Planctomycetales | Planctomycetaceae | Gemmata | 1 |
| Planctomycetes | Planctomycetales | Planctomycetaceae | Gemmata | 1 |
| Planctomycetes | Planctomycetales | Planctomycetaceae | Pirellula | 1 |
| Planctomycetes | Planctomycetales | Planctomycetaceae | Pirellula | 1 |
| Planctomycetes | Planctomycetales | Planctomycetaceae | Singulisphaera | 1 |
| Planctomycetes | Planctomycetales | Planctomycetaceae | Thermogutta | 1 |
| Planctomycetes | Planctomycetales | Planctomycetaceae | Zavarzinella | 1 |
| Proteobacteria | Alphaproteobacteria_incertae_sedis | Geminicoccus | Geminicoccus | 1 |
| Proteobacteria | Burkholderiales | Oxalobacteraceae | Massilia | 1 |
| Proteobacteria | Burkholderiales | Oxalobacteraceae | Massilia | 1 |
| Proteobacteria | Caulobacterales | Caulobacteraceae | Caulobacter | 1 |
| Proteobacteria | Myxococcales | Kofleriaceae | Kofleria | 1 |
| Proteobacteria | Myxococcales | Polyangiaceae | Sorangium | 1 |
| Proteobacteria | Oceanospirillales | Oceanospirillales_incertae_sedis | Pseudohongiella | 1 |
| Proteobacteria | Pseudomonadales | Pseudomonadaceae | Pseudomonas | 1 |
| Proteobacteria | Pseudomonadales | Pseudomonadaceae | Pseudomonas | 1 |
| Proteobacteria | Rhizobiales | Bradyrhizobiaceae | Bradyrhizobium | 1 |
| Proteobacteria | Rhizobiales | Hyphomicrobiaceae | Devosia | 1 |
| Proteobacteria | Rhizobiales | Hyphomicrobiaceae | Devosia | 1 |
| Proteobacteria | Rhizobiales | Methylobacteriaceae | Methylobacterium | 1 |
| Proteobacteria | Rhizobiales | Methylobacteriaceae | Microvirga | 1 |
| Proteobacteria | Rhizobiales | Methylobacteriaceae | Microvirga | 1 |
| Proteobacteria | Rhizobiales | Methylobacteriaceae | Microvirga | 1 |
| Proteobacteria | Rhizobiales | Phyllobacteriaceae | Chelativorans | 1 |
| Proteobacteria | Rhizobiales | Rhizobiales_incertae_sedis | Vasilyevaea | 1 |
| Proteobacteria | Rhodobacterales | Rhodobacteraceae | Rubellimicrobium | 1 |
| Proteobacteria | Rhodobacterales | Rhodobacteraceae | Rubellimicrobium | 1 |
| Proteobacteria | Rhodospirillales | Acetobacteraceae | Belnapia | 1 |
| Proteobacteria | Rhodospirillales | Acetobacteraceae | Belnapia | 1 |
| Proteobacteria | Rhodospirillales | Acetobacteraceae | Belnapia | 1 |
| Proteobacteria | Rhodospirillales | Acetobacteraceae | Belnapia | 1 |
| Proteobacteria | Rhodospirillales | Acetobacteraceae | Craurococcus | 1 |
| Proteobacteria | Rhodospirillales | Acetobacteraceae | Roseococcus | 1 |
| Proteobacteria | Rhodospirillales | Acetobacteraceae | Roseomonas | 1 |
| Proteobacteria | Rhodospirillales | Rhodospirillaceae | Skermanella | 1 |
| Proteobacteria | Sphingomonadales | Sphingomonadaceae | Sphingomonas | 1 |
| Proteobacteria | Sphingomonadales | Sphingomonadaceae | Sphingomonas | 1 |
| Proteobacteria | Sphingomonadales | Sphingomonadaceae | Sphingomonas | 1 |
| Proteobacteria | Xanthomonadales | Sinobacteraceae | Povalibacter | 1 |
| Proteobacteria | Xanthomonadales | Sinobacteraceae | Steroidobacter | 1 |
| Proteobacteria | Xanthomonadales | Xanthomonadaceae | Dokdonella | 1 |
| Proteobacteria | Xanthomonadales | Xanthomonadaceae | Rhodanobacter | 1 |
| Verrucomicrobia | Spartobacteria_genera_incertae_sedis | Spartobacteria_genera_incertae_sedis | Spartobacteria_genera_incertae_sedis | 1 |
| Verrucomicrobia | Spartobacteria_genera_incertae_sedis | Spartobacteria_genera_incertae_sedis | Spartobacteria_genera_incertae_sedis | 1 |
| Verrucomicrobia | Spartobacteria_genera_incertae_sedis | Spartobacteria_genera_incertae_sedis | Spartobacteria_genera_incertae_sedis | 1 |
| Verrucomicrobia | Spartobacteria_genera_incertae_sedis | Spartobacteria_genera_incertae_sedis | Spartobacteria_genera_incertae_sedis | 1 |
| Verrucomicrobia | Subdivision3_genera_incertae_sedis | Subdivision3_genera_incertae_sedis | Subdivision3_genera_incertae_sedis | 1 |
| Verrucomicrobia | Subdivision3_genera_incertae_sedis | Subdivision3_genera_incertae_sedis | Subdivision3_genera_incertae_sedis | 1 |

**Supplementary Table 6.** Fungal taxonomy in the soil microbial community used for the inoculation treatment. Taxa are ordered by count and then alphabetically by phyla, class, order, family, and genus.

| **Phyla** | **Class** | **Order** | **Family** | **Genus** | **Count** |
| --- | --- | --- | --- | --- | --- |
| Ascomycota | Dothideomycetes | Pleosporales | Pleosporaceae | Alternaria | 1123 |
| Ascomycota | Dothideomycetes | Pleosporales | Pleosporaceae | Pleospora | 335 |
| Basidiomycota | Agaricomycetes | Cantharellales | Ceratobasidiaceae | Thanatephorus | 314 |
| Ascomycota | Dothideomycetes | Pleosporales | Sporormiaceae | Westerdykella | 223 |
| Ascomycota | Sordariomycetes | Coniochaetales | Coniochaetaceae | Coniochaeta | 161 |
| Ascomycota | Dothideomycetes | Pleosporales | Sporormiaceae | Westerdykella | 124 |
| Ascomycota | Dothideomycetes | Capnodiales | Davidiellaceae | Davidiella | 119 |
| Ascomycota | Sordariomycetes | Sordariales | Chaetomiaceae | Chaetomidium | 115 |
| Ascomycota | Dothideomycetes | Pleosporales | Montagnulaceae | Karstenula | 104 |
| Ascomycota | Dothideomycetes | Pleosporales | Pleosporaceae | Alternaria | 70 |
| Ascomycota | Pezizomycetes | Pezizales | Pyronemataceae | Marcelleina | 61 |
| Basidiomycota | Agaricomycetes | Agaricales | Agaricaceae | Tulostoma | 61 |
| Ascomycota | Dothideomycetes | Pleosporales | Sporormiaceae | Preussia | 57 |
| Ascomycota | Dothideomycetes | Pleosporales | Lophiostomataceae | Lophiostoma | 53 |
| Basidiomycota | Tremellomycetes | Cystofilobasidiales | Cystofilobasidiaceae | Udeniomyces | 49 |
| Ascomycota | Sordariomycetes | Hypocreales | Niessliaceae | Emericellopsis | 43 |
| Basidiomycota | Agaricomycetes | Agaricales | Pluteaceae | Volvariella | 40 |
| Ascomycota | Dothideomycetes | Pleosporales | Phaeosphaeriaceae | Phaeodothis | 37 |
| Ascomycota | Dothideomycetes | Pleosporales | Pleosporaceae | Stagonospora | 37 |
| Ascomycota | Dothideomycetes | Capnodiales | Capnodiales incertae sedis | Penidiella | 32 |
| Basidiomycota | Agaricomycetes | Geastrales | Geastraceae | Geastrum | 32 |
| Ascomycota | Sordariomycetes | Hypocreales | Hypocreaceae | Trichoderma | 22 |
| Ascomycota | Sordariomycetes | Sordariales | Chaetomiaceae | Chaetomidium | 22 |
| Basidiomycota | Agaricomycetes | Agaricales | Agaricaceae | Tulostoma | 19 |
| Ascomycota | Sordariomycetes | Hypocreales | Nectriaceae | Gibberella | 15 |
| Chytridiomycota | Chytridiomycetes | Spizellomycetales | Spizellomycetaceae | Rhizophlyctis | 13 |
| Ascomycota | Dothideomycetes | Pleosporales | Sporormiaceae | Westerdykella | 11 |
| Chytridiomycota | Chytridiomycetes | Spizellomycetales | Spizellomycetaceae | Rhizophlyctis | 11 |
| Ascomycota | Dothideomycetes | Pleosporales | Phaeosphaeriaceae | Phaeosphaeria | 10 |
| Ascomycota | Dothideomycetes | Pleosporales | Pleosporaceae | Alternaria | 7 |
| Ascomycota | Pezizomycetes | Pezizales | Pyronemataceae | Geopora | 5 |
| Ascomycota | Sordariomycetes | Diaporthales | Valsaceae | Phomopsis | 5 |
| Basidiomycota | Agaricomycetes | Agaricales | Agaricaceae | Tulostoma | 5 |
| Ascomycota | Dothideomycetes | Pleosporales | Sporormiaceae | Westerdykella | 4 |
| Ascomycota | Sordariomycetes | Microascales | Microascaceae | Doratomyces | 4 |
| Ascomycota | Dothideomycetes | Pleosporales | Phaeosphaeriaceae | Phaeosphaeria | 3 |
| Ascomycota | Dothideomycetes | Pleosporales | Sporormiaceae | Westerdykella | 2 |
| Ascomycota | Eurotiomycetes | Eurotiales | Trichocomaceae | Emericella | 1 |
| Ascomycota | Pezizomycetes | Pezizales | Pezizaceae | Iodophanus | 1 |
| Ascomycota | Sordariomycetes | Hypocreales | Clavicipitaceae | Paecilomyces | 1 |
| Ascomycota | Sordariomycetes | Hypocreales | Hypocreaceae | Hypocrea | 1 |
| Ascomycota | Sordariomycetes | Hypocreales | Nectriaceae | Neocosmospora | 1 |
| Ascomycota | Sordariomycetes | Sordariales | Chaetomiaceae | Chaetomidium | 1 |
| Ascomycota | Sordariomycetes | Sordariales | Chaetomiaceae | Chaetomium | 1 |
| Basidiomycota | Tremellomycetes | Tremellales | Sirobasidiaceae | Fibulobasidium | 1 |

**Supplementary Figure 1.** Representative pressure-volume curves for each family and treatment where the inverse of leaf water potential (1/ψ) is plotted against relative water deficit (RWD).
